# Rapid Shifts in Mitochondrial tRNA Import in a Plant Lineage with Extensive Mitochondrial tRNA Gene Loss

**DOI:** 10.1093/molbev/msab255

**Published:** 2021-08-26

**Authors:** Jessica M Warren, Thalia Salinas-Giegé, Deborah A Triant, Douglas R Taylor, Laurence Drouard, Daniel B Sloan

**Affiliations:** 1 Department of Biology, Colorado State University, Fort Collins, CO, USA; 2 Institut de Biologie Moléculaire des Plantes-CNRS, Université de Strasbourg, Strasbourg, France; 3 Department of Biochemistry & Molecular Genetics, University of Virginia, Charlottesville, VA, USA; 4 Department of Biology, University of Virginia, Charlottesville, VA, USA

**Keywords:** tRNA import, tRNA-seq, mitochondrial tRNAs, plants, angiosperms

## Abstract

In most eukaryotes, transfer RNAs (tRNAs) are one of the very few classes of genes remaining in the mitochondrial genome, but some mitochondria have lost these vestiges of their prokaryotic ancestry. Sequencing of mitogenomes from the flowering plant genus *Silene* previously revealed a large range in tRNA gene content, suggesting rapid and ongoing gene loss/replacement. Here, we use this system to test longstanding hypotheses about how mitochondrial tRNA genes are replaced by importing nuclear-encoded tRNAs. We traced the evolutionary history of these gene loss events by sequencing mitochondrial genomes from key outgroups (*Agrostemma githago* and *Silene* [=*Lychnis*] *chalcedonica*). We then performed the first global sequencing of purified plant mitochondrial tRNA populations to characterize the expression of mitochondrial-encoded tRNAs and the identity of imported nuclear-encoded tRNAs. We also confirmed the utility of high-throughput sequencing methods for the detection of tRNA import by sequencing mitochondrial tRNA populations in a species (*Solanum tuberosum*) with known tRNA trafficking patterns. Mitochondrial tRNA sequencing in *Silene* revealed substantial shifts in the abundance of some nuclear-encoded tRNAs in conjunction with their recent history of mt-tRNA gene loss and surprising cases where tRNAs with anticodons still encoded in the mitochondrial genome also appeared to be imported. These data suggest that nuclear-encoded counterparts are likely replacing mitochondrial tRNAs even in systems with recent mitochondrial tRNA gene loss, and the redundant import of a nuclear-encoded tRNA may provide a mechanism for functional replacement between translation systems separated by billions of years of evolutionary divergence.

## Introduction

The existence of multiple genomes within eukaryotic cells necessitates multiple gene expression systems. Protein synthesis occurs separately in endosymbiotically derived organelles (mitochondria and plastids) and the cytosol. The coding capacity of mitochondrial genomes (mitogenomes) has dwindled from estimated thousands of genes in the mitochondrial progenitor to only a few dozen in most eukaryotes (Gray 2012). The import of gene products encoded in the nuclear genome compensates for many of these organellar gene losses (Huynen et al. 2013). Yet despite their reduced gene content, almost all mitogenomes still encode at least some components of the translational machinery (Sloan et al. 2018).

Clear patterns have emerged in the retention and loss of certain genes involved in organellar translation systems. The genes encoding the large and small subunit mitochondrial ribosomal RNAs (rRNAs) are almost universally retained in the mitogenome, whereas other gene products, such as aminoacyl tRNA synthetases (aaRSs), are exclusively encoded in the nuclear genome and must be imported into the mitochondrial matrix (Sissler et al. 2005). The genes for transfer RNAs (tRNAs) exhibit much more heterogeneity concerning their retention in mitogenomes.

A set of 22 mt-tRNA genes that is sufficient to decode all codons has been retained for over 500 million years in some animal mitogenomes (Anderson et al. 1981; Clary and Wolstenholme 1984; Peterson et al. 2004), but the sampling of mitogenomes from diverse taxonomic groups has revealed extensive variation in complements of mt-tRNAs with examples of extreme mt-tRNA loss. Some Trypanosomatids (Simpson et al. 1989; Hancock and Hajduk 1990) and the lycophyte *Selaginella moellendorffii* (Hecht et al. 2011) entirely lack tRNA genes in their respective mitogenomes, whereas some bilaterian animals only have a single mt-tRNA gene (Lavrov and Pett 2016). In contrast, flowering plants have an intermediate and heterogeneous set of mt-tRNA genes. Their mitogenomes typically have 11–13 native (mitochondrial in origin) tRNA genes in addition to a variable number of intracellularly and horizontally transferred tRNAs from diverse origins including plastids, bacteria, fungi, and the mitochondria of other plants (Iams et al. 1985; Small et al. 1999; Kubo et al. 2000; Rice et al. 2013; Knie et al. 2015; Sanchez-Puerta et al. 2019; Warren and Sloan 2020). Despite this mosaic of mt-tRNA genes, no angiosperm species appears to have a mitochondrially encoded tRNA for every amino acid (Michaud et al. 2011).

The import of nuclear-encoded tRNAs in mitochondria has been demonstrated in a handful of plant species and is assumed to compensate for the insufficient coding capacity of mt-tRNAs in the mitogenome (Ceci et al. 1995; Kumar et al. 1996; Brubacher-Kauffmann et al. 1999; Duchêne and Marechal-Drouard 2001; Glover et al. 2001). Because most plants still retain many mt-tRNA genes, they require only a specific subset of imported tRNAs for mitochondrial function. The import of nuclear-encoded tRNAs into plant mitochondria from the cytosol is complementary rather than redundant, meaning that tRNAs corresponding to the genes functionally lost from the mitogenome are selectively imported (Kumar et al. 1996; Brubacher-Kauffmann et al. 1999; Salinas-Giegé et al. 2015). This specificity of cytosolic tRNA import even extends beyond the anticodon level within isoacceptor families, as tRNA-Gly transcripts are differentially imported or excluded in *Solanum tuberosum* mitochondria depending on the anticodon (Brubacher-Kauffmann et al. 1999). In addition, tRNA import is associated with translation optimization and codon usage in the green alga *Chlamydomonas reinhardtii* (Vinogradova et al. 2009).

**Fig. 1.**
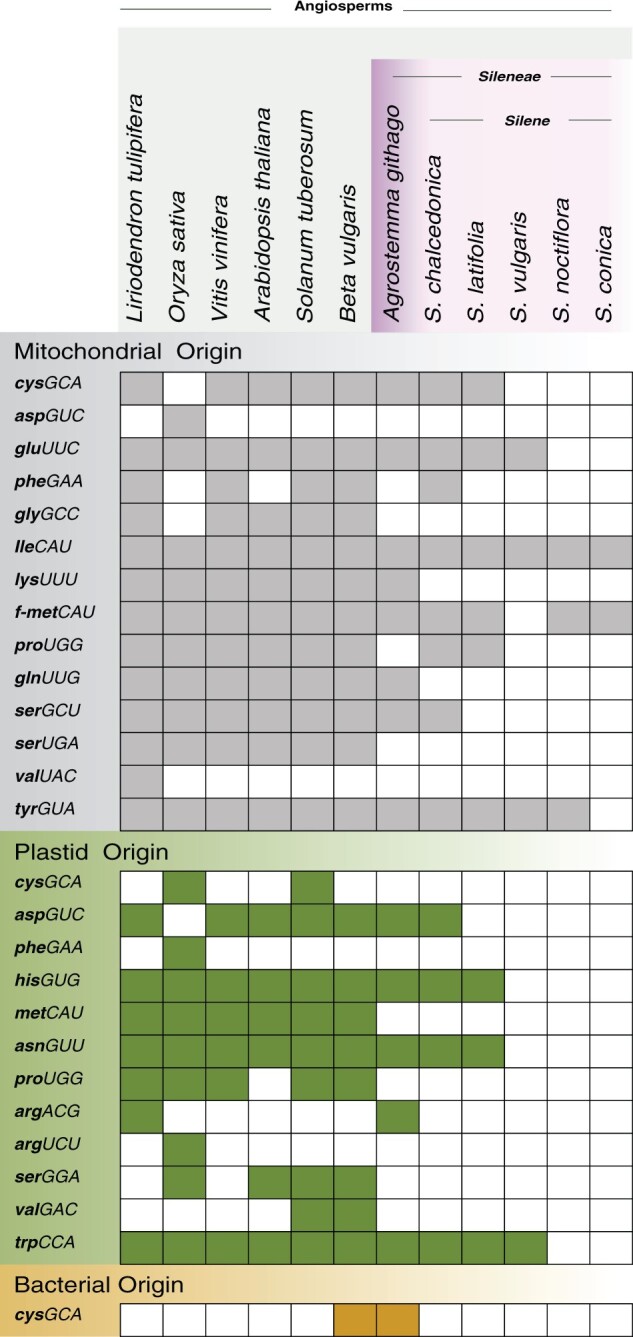
Summary of tRNA gene content in a sample of angiosperm mitogenomes with an emphasis on the family Caryophyllaceae. Filled squares indicate the presence of an intact gene sequence, with genes named based on the three-letter amino acid code in lowercase and the anticodon in uppercase. Formyl-methionine is abbreviated f-met. Different colors represent the ancestral origin of the tRNA gene. Disrupted or incomplete sequences inferred to be pseudogenes are not included.

Heterogeneity in mt-tRNA content appears to arise over short evolutionary timeframes in plants, raising questions about the evolutionary dynamics of functional replacement of tRNAs in light of observed tRNA import specificity. The sequencing of mitogenomes from the angiosperm genus *Silene* revealed multiple species with greatly reduced numbers of tRNA genes (fig. 1) (Sloan, Alverson, Chuckalovcak, et al. 2012). Many of these mitochondrial gene losses appear to have happened recently as the closely related species *S. latifolia*, *S. vulgaris*, *S. noctiflora*, and *S. conica* have nine, four, three, and two mt-tRNA genes respectively (fig. 1). These four species represent different sections within the same subgenus and have been difficult to resolve phylogenetically (Jafari et al. 2020), making their precise history of shared and independent tRNA-gene losses unclear.

This rapid and ongoing loss of mt-tRNAs in *Silene* presents a unique opportunity to study the evolutionary mechanisms involved in the functional replacement of mt-tRNAs with nuclear-encoded counterparts. However, characterizing the mt-tRNA pools (including both imported and mitochondrially encoded tRNAs) presents numerous challenges—the first being the intrinsic difficulty in sequencing tRNAs. The vast majority of RNA-seq methods require the reverse transcription of the molecules before sequencing, but extensive base modifications in tRNAs can stall or terminate reverse transcriptase enzymes (Wilusz 2015). Additionally, the tightly base-paired 5′ and 3′ termini of tRNAs can prevent the ligation of sequencing adapters (Shigematsu et al. 2017). To date, global sequencing of purified mt-tRNA pools has only been done in humans (Mercer et al. 2011). The second challenge in mt-tRNA genetics arises from the degenerate nature of mt-tRNAs. Although mt-tRNAs in plants are predicted to have a generally canonical shape (i.e., a cloverleaf-shape with a D-arm, a T-arm, an acceptor stem, an anticodon stem, and associated loops [Raina and Ibba 2014]), mt-tRNAs from other lineages (particularly metazoans) can have such aberrant structure that determining the number and location of mt-tRNA genes is unreliable using only genomic data and predictive software (Boore et al. 2005; Juhling et al. 2009). For example, in some mite species, the majority of expressed mt-tRNAs were not detected by tRNAscan when the mitogenome was searched (Warren and Sloan 2021). The existence of degenerate mt-tRNAs in some lineages raises the possibility that putatively lost tRNA genes in *Silene* are still present in the mitogenome but simply undetected. A third challenge is that the majority of tRNA import research in plants has relied on northern blot analysis (Kumar et al. 1996; Delage et al. 2003). This approach requires previously predicted tRNA sequences for hybridization and can lack the clear resolution of probed sequences because of cross-hybridization to undesired targets. Therefore, multiple questions remain about the composition of tRNA populations in the plant mitochondrial matrix.

Here, we trace the evolutionary history of a plant lineage with extreme mt-tRNA loss using recently developed tRNA-seq methods and mitogenome sequencing. We provide the first global sequencing of tRNA pools from isolated plant mitochondria, characterizing the expression of mt-tRNA genes as well the population of imported cytosolic sequences. Using these analyses, we address the following questions: First, have *Silene* mitogenomes really experienced an extreme reduction in mt-tRNA gene content, or do they contain “hidden” genes that are unrecognizable with standard annotation methods? If mt-tRNA genes have been lost and functionally replaced by the import of cytosolic tRNAs, are certain nuclear-encoded tRNAs dedicated to mitochondrial translation? And finally, do *Silene* species preserve the import specificity that has been demonstrated in other plant species, maintaining the avoidance of functionally redundant tRNA import?

This study supports the conclusion from previous mitogenome annotations that *Silene* species have experienced extensive mt-tRNA gene loss and reveals widespread shifts in the abundance of certain nuclear-encoded tRNAs in isolated mitochondria across *Sileneae* species—suggesting that import of tRNAs from the cytosol compensates for mt-tRNA gene losses. Additionally, the functional replacement of mt-tRNAs with nuclear-encoded counterparts does not appear to involve the expression and import of tRNA genes exclusively dedicated for mitochondrial function. Finally, we show multiple instances where tRNAs with anticodons still encoded in the mitogenome appear to be imported in *Sileneae*, indicating a loss of the tRNA import specificity previously seen in other plant mitochondria.

## Results

### Tracing the Evolution of mt-tRNA Gene Loss in *Sileneae* by Sequencing the Mitochondrial Genomes of *Agrostemma githago* and *Silene chalcedonica*

All previously sequenced *Silene* mitogenomes have been found to have extensive mt-tRNA gene loss (Sloan, Alverson, Chuckalovcak, et al. 2012); however, all of the species that have been sequenced are from a single subgenus, *Behenantha*. In order to better reconstruct the history and timing of mt-tRNA gene losses, we expanded mitogenome sampling by sequencing a representative of the subgenus *Lychnis* (*Silene chalcedonica*) (Jafari et al. 2020), as well as *Agrostemma githago*, which is also a member of the tribe *Sileneae* (fig. 1). Prior to this work, the closest sequenced relative to the genus *Silene* was *Beta vulgaris* (sugar beet), representing an estimated 70 Myr of divergence from *Silene* (Magallon et al. 2015). The mitogenome of *B. vulgaris* has a more typical set of 19 mt-tRNA genes (Kubo et al. 2000) and lacks the history of gene loss seen in *Silene*.

The assembly of the *A. githago* mitogenome produced a 262,903 bp master circle with a GC content of 44.7%. The assembly included four copies of a 2,427 bp “core” repeat region, with additional repeated sequence flanking this core region but only present in a subset of the four copies. This structure of large, identical repeat regions is similar to the mitogenome of *S. latifolia*, which has six copies of a (different) large repeat (Sloan et al. 2010). These repeats likely undergo intra- and intermolecular recombination, resulting in multiple genome configurations not depicted by the master circle represented here (supplementary fig. 1, Supplementary Material online).

The protein-coding gene inventory of *A. githago* is similar to that of members of the genus *Silene* (supplementary fig. 1, Supplementary Material online). Interestingly, the intron-containing gene *nad7* appears to be *trans*-spliced in *A. githago*. While the first four exons are found in a standard *cis* configuration, the fifth exon is located elsewhere in the genome (supplementary fig. 1, Supplementary Material online). Other NADH-ubiquinone oxidoreductase genes (specifically *nad1*, *nad2*, and *nad5*) typically contain *trans*-splicing introns (Chapdelaine and Bonen 1991; Knoop et al. 1991; Wissinger et al. 1991; Binder et al. 1992), but this is not the case for *nad7* in most angiosperm mitogenomes. Such transitions from *cis*- to *trans*-splicing are relatively common in plant mitochondria (Qiu and Palmer 2004), and it was recently shown that the Pinaceae has independently evolved *trans*-splicing of this same *nad7* intron (Guo et al. 2020).

The complement of 14 mt-tRNA genes in *A. githago* also falls below the typical number encoded in angiosperm mitogenomes, but it is greater than the previously documented mt-tRNA gene content in *Silene* (fig. 1), putting *A. githago* at an intermediate state of mt-tRNA gene loss. There is, however, evidence of independent mt-tRNA gene loss in *A. githago* as both *pheGAA* and *proUGG* have been lost in *A. githago* but they are present in at least one *Silene* species (fig. 1, tRNA genes are indicated in italic with the three-letter amino acid code in lowercase and the anticodon in uppercase). Two distinct copies of *cysGCA* are present in *A. githago*: the native mitochondrial copy and the bacterial, horizontally transferred gene that was first reported in the mitogenome of *B. vulgaris* (Kubo et al. 2000; Kitazaki et al. 2011) (fig. 1 and supplementary table 1, Supplementary Material online). The native *glyGCC* and *serUGA*, as well as the plastid-derived elongator *metCAU* and *serGGA*, appear to have been lost before the divergence of *Agrostemma* and *Silene* as they are absent from *A. githago* and all currently sequenced *Silene* species (fig. 1). As *A. githago* does have reduced mt-tRNA gene content compared to most angiosperms, the process of tRNA gene loss likely initiated prior to the divergence of *Sileneae*, but it is has proceeded to much greater extents in the sampled species from *Silene* subgenus *Behenantha*.

The mitogenome of *S. chalcedonica* was found to be considerably larger than that of *A. githago* with an estimated size of approximately 880 kb and a far more complex repeat structure. Because large, highly repetitive genomes are difficult to “close,” we did not try to produce a master circle assembly for the species. However, sequencing resulted in high coverage (>50× on average) of assembled mitochondrial contigs, suggesting that we likely captured all sequence content for the identification of mt-tRNA genes. *Silene chalcedonica* was found to have 12 predicted mt-tRNA genes (two fewer than in *A. githago*). Multiple tRNA gene losses are shared by other *Silene* species (fig. 1). For example, *glnUUG* was likely lost before the divergence of *S. chalcedonica* and the other *Silene* species, as none of the sequenced members of the genus have an intact copy of the gene. However, *serGCU* and *pheGAA* are still present in *S. chalcedonica*, whereas they have been lost in the other sequenced *Silene* species (fig. 1).

### Confirming the Expression of Mitochondrially Encoded tRNA Genes in *Sileneae* Species

To validate the *in silico* annotations of mitochondrially encoded tRNA genes and characterize global mt-tRNA populations, mitochondria were isolated from leaf tissue of five *Sileneae* species (*A. githago*, *S. conica*, *S. latifolia*, *S. noctiflora*, and *S. vulgaris*) using differential centrifugation and discontinuous Percoll gradients. Paired, total-cellular RNA (whole leaf tissue) samples were processed in parallel with each mitochondrial sample. tRNAs were then sequenced with recently developed tRNA-seq protocols including treatment with the dealkylating enzyme AlkB to remove base modifications that inhibit reverse transcription (Cozen et al. 2015; Zheng et al. 2015) and the use of adapters with complementarity to the CCA-tail found on all mature tRNAs (Shigematsu et al. 2017). Sequences were mapped to annotated mt-tRNA genes, as well as the entire mitogenome of each respective species to search for previously undetected tRNAs. Reads were also mapped to tRNA genes predicted from nuclear assemblies of the five species by tRNAscan-SE (Chan and Lowe 2019).

**Fig. 2.**
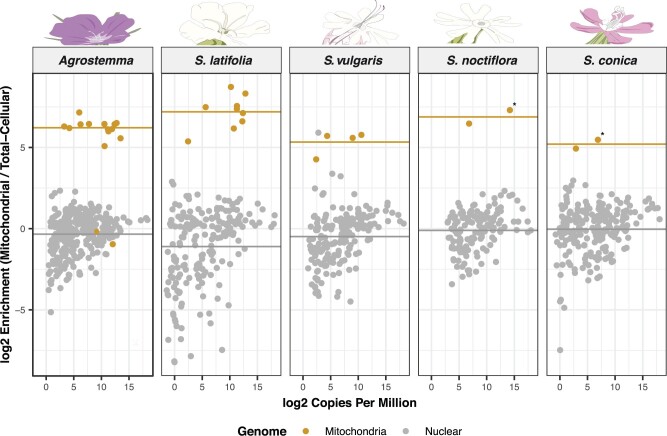
Enrichment of mitochondrially encoded and nuclear-encoded tRNAs in mitochondrial isolates relative to total-cellular samples. Each dot represents a unique tRNA sequence. The heavy gray line represents the average enrichment of nuclear-encoded genes. The genomic origin of the tRNA is indicated by color, with gray being nuclear-encoded and gold being mitochondrial. The two mt-tRNAs around the 0-line for *Agrostemma* are tRNA-Asn(GUU) and tRNA-Arg(ACG), which are identical in sequence to the plastid-encoded tRNA counterparts and were not included in the calculation of average enrichment of mitochondrially encoded tRNAs for the species. Expression of the multiple (but nonidentical) copies of formyl-methionine (fMet) tRNAs present in in the mitogenomes of *S. noctiflora* and *S. conica* are marked with an * and were summed for expression analysis. CCA-tailed stem-loops identified in this study are not shown in this figure.

As described in detail below, the purified mitochondrial samples showed strong enrichment for mitochondrially encoded tRNAs (fig. 2), confirming the efficacy of the mitochondrial isolations. All mt-tRNA genes previously predicted to be functional in *S. conica*, *S. latifolia*, *S. noctiflora*, and *S. vulgaris* were found to be expressed (supplementary table 2, Supplementary Material online). Two *A. githago* mt-tRNAs (tRNA-Asn[GUU], and tRNA-Arg[ACG]) with sequences that are identical to plastid copies were depleted in mitochondrial isolates (supplementary table 3, Supplementary Material online), suggesting that these tRNAs may not be functional in the mitochondrial matrix (specific tRNAs are indicated with a nonitalicized three-letter amino acid code and the anticodon in parentheses). Both tRNAs have 100% sequence identity to plastid tRNA genes that are present within larger plastid-derived sequence blocks (i.e., an “mtpt”), indicating that these sequences are recent insertions of plastid DNA. The *asnGUU* gene is likely different from the ancestral plastid-derived *asnGUU* copy found in most angiosperm mitogenomes (fig. 1), which can be differentiated from plastid copies by mitochondrial-specific sequence variants (Richardson et al. 2013). The lack of detection of both genes suggests that that reduction in mt-tRNA gene content in *A. githago* may be more extreme than apparent from genomic data alone. Differential expression (enrichment) analysis for all mitochondrial genes can be found for each species in supplementary tables 3–8, Supplementary Material online.

### The Post-Transcriptional Addition of a CCA Tail Is Widespread in Sileneae Mitochondria and Occurs on Multiple Non-tRNA Transcripts

Although the vast majority of reads from the mitogenome mapped to an annotated tRNA gene, a small percentage mapped to other positions (table 1 and supplementary table 9, Supplementary Material online). These reads frequently had CCA nucleotides at a terminus and occurred at the boundaries of rRNA or protein-coding genes (supplementary table 9, Supplementary Material online). For some of these sequences, the CCA nucleotides appear to be post-transcriptionally added as the sequence is not genomically encoded. Many of these reads formed stem-loop structures that act as t-element processing signals previously described in other angiosperms (Forner et al. 2007; Varre et al. 2019)*.* T-elements are tRNA-like sequences bordering other genes that act as signals for endonucleolytic cleavage by RNase Z and/or RNase P for mRNA or rRNA transcript maturation (Forner et al. 2007). For example, a conserved t-element structure internal to the annotated *nad6* open reading frame was expressed and post-transcriptionally modified with a CCA-tail in *A. githago*, *S. latifolia*, and *S. vulgaris* (supplementary table 9 and fig. 2, Supplementary Material online).

**Table 1.** Percentage of Reads with a CCA-Tail That Mapped to a Mitochondrial Location Other Than a tRNA Gene.

Species	Mitochondrial Isolate 1	Mitochondrial Isolate 2	Mitochondrial Isolate 3	Total Cellular 1	Total Cellular 2	Total Cellular 3
*Agrostemma*	0.2316%	0.3290%	0.2057%	0.0085%	0.0072%	0.0086%
*S. latifolia*	0.0700%	0.0438%	NA	0.0009%	0.0003%	NA
*S. vulgaris*	4.4819%	NA	3.6261%	0.1084%	0.1553%	0.3070%
*S. noctiflora*	0.0243%	0.0314%	0.0234%	0.0012%	0.0018%	0.0059%
*S. conica*	0.0005%	0.0008%	0.0014%	0.0000%	0.0000%	0.0000%

Only sequences that had more than two reads (in all six libraries combined) were used for global mitogenome mapping and this analysis. Only two replicates of *S. latifolia* mitochondrial isolations, *S. latifolia* total-cellular samples, and *S. vulgaris* mitochondrial isolations were performed; see Materials and Methods for details.

Although most of these stem-loops lacked canonical tRNA structure, they did have stem structure at the 5′- and 3′-termini (supplementary fig. 2, Supplementary Material online). The CCA-tailing of stem-loop structures has been reported in numerous other mitochondrial systems including rats (Yu et al. 2008), humans (Pawar et al. 2019), mites (Warren and Sloan 2021), and other plants (Williams et al. 2000). Interestingly, the species with the fewest mt-tRNAs (*S. conica* and *S. noctiflora*) also had the fewest of these detected stem-loops (table 1, supplementary table 9, Supplementary Material online). Only 0.0005–0.0314% of the CCA-containing reads in these two species mapped to a non-tRNA position in the mitogenome, and these reads almost exclusively originated from the boundaries of rRNA genes (supplementary table 9, Supplementary Material online). In comparison, the percentage of reads mapping to non-tRNA position in the mitogenomes in the other three species ranged from 0.0438% up to 4.4819%. The relatively abundant reads mapping to a stem-loop structure in *S. vulgaris* derived mainly from a region of the mitogenome with two identical step-loop sequences that were not immediately up- or down-stream of an annotated gene (over 6 kb away from *cob* and over 4.5 kb away from the fourth exon of *nad5*). *Silene vulgaris* also had a relatively highly abundant stem-loop structure originating from an *f-metCAU* gene (over 51,000 reads combined in all six libraries). This sequence is annotated as a pseudogene as it lacks multiple features of a tRNA, and it is predicted to form an arm-less, degenerate stem-loop structure with multiple bulges (supplementary fig. 2, Supplementary Material online). It is only 58 bp away from *atp6*, so it is likely that it now serves as a t-element maturation signal.

One stem-loop structure mapped to the mitogenome of *A. githago* did have a cloverleaf-like structure similar to a canonical tRNA shape but did not correspond to an annotated tRNA gene (supplementary fig. 3 and table 2*a*, Supplementary Material online). Predictive folding under a maximum free energy model puts the anticodon as tRNA-Lys(UUU) (supplementary fig. 3, Supplementary Material online). Unlike t-elements, this stem-loop is not closely associated with any annotated gene, as it is 2,414 bp upstream of the nearest gene (the fourth exon of *nad1*, supplementary table 9, Supplementary Material online). Homologous sequences are present in the mitogenome of *Beta vulgaris* (GenBank: BA000024) and *Chenopodium quinoa* (GenBank: NC_041093), as well as copies located within a region of mitochondrial-like sequence in the nuclear genome assembly of *Linum usitatissimum* (GenBank: CP027627). However, this sequence is not present in the mitogenomes of any of the sequenced *Silene* species. Although the evolution of novel tRNAs through the duplication of existing tRNA genes has been shown phylogenetically and experimentally (Ayan et al. 2020), this sequence in *Agrostemma* does not have identifiable homology to any known tRNA and does not appear to form a tRNA-like structure in *B. vulgaris* or *C. quinoa*. Whether this is just a nonfunctional stem-loop that happened to converge on a predicted cloverleaf structure or a functional decoding molecule representing the *de novo* birth of a tRNA gene is not clear.

**Table 2.** Depletion of tRNAs from the Nuclear and Plastid Genomes in Mitochondrial Isolates from Six Angiosperm Species Relative to Mitochondrially Encoded tRNAs.

	Average Plastid tRNA Depletion	Average Nuclear tRNA Depletion^a^
Species	Relative log_2_ fold difference	Relative log_2_ fold difference
*Solanum*	−4.43	−4.75
*Agrostemma*	−6.76	−6.55
*S. latifolia*	−7.56	−8.57
*S. vulgaris*	−6.37	−5.78
*S. noctiflora*	−8.68	−6.99
*S. conica*	−7.45	−4.88

Notes.—Nuclear tRNA depletion was calculated as the difference between the average log_2_ fold change of all mt-tRNA genes (including mitochondrial stem-loops) and the average log_2_ fold change of nuclear-encoded tRNAs. Average plastid tRNA depletion was calculated similarly as the difference between the average mt-tRNA log_2_ fold change and the average log_2_ fold change of plastid tRNAs.

a Note that the depletion values for nuclear-encoded tRNAs do not account for the fact that some of these tRNAs are presumably imported into the mitochondria. Thus, the values reported here likely underestimate the true depletion levels for nonimported, nuclear-encoded tRNAs.

For multiple reasons, it is unlikely that the vast majority of the “stem-loops” detected in this data set represent functional tRNAs. First, with the exception of the tRNA-like-Lys(UUU) structure in *A. githago*, these stem-loops have structural deformities that would be generally inconsistent with tRNA function, including multiple mismatches, a lack of a cloverleaf or L-shape, and incorrectly sized anticodon loops. Even in extremely aberrant metazoan mt-tRNAs that lack arms, an L-shape tertiary structure is still expected (Watanabe et al. 2014; Juhling et al. 2018). Mismatched bases in stems of plant mt-tRNAs have been predicted from genomic data but found to be corrected post-transcriptionally (Marechal-Drouard et al. 1993; Binder et al. 1994). The sequenced stem loops found in this study maintained these base mismatches. Secondly, many of these structures originate from known t-element regions or are directly up or downstream of other genes. T-elements are broadly present in angiosperms (including those with much larger mt-tRNA gene sets) and are known substrates for other tRNA-processing machinery (i.e., RNase P/Z) (Forner et al. 2007). Thus, it is likely that these sequences are just processed with a CCA-tail as a byproduct. Lastly, these stem-loop sequences were of low abundance and almost absent in the species with the fewest mt-tRNA genes. Therefore, it does not appear that the apparent mt-tRNA gene loss seen in *Sileneae* species can be explained by a large class of previously undetected tRNA genes in the mitogenome.

### Characterization of Imported tRNA Pools in *Sileneae* Mitochondria Reveals Extensive Sharing of the Same Nuclear-Encoded tRNAs between the Cytosol and the Mitochondria

The average enrichment of mitochondrial-encoded tRNAs in purified *Sileneae* mitochondrial fractions was anywhere from 37- to 146-fold relative to total-cellular samples (supplementary tables 3–8, Supplementary Material online) depending on the species. Almost no nuclear-encoded tRNAs in any of the five *Sileneae* species reached the enrichment level of the mitochondrially encoded tRNAs (fig. 2), suggesting that very few if any, nuclear-encoded tRNAs exclusively function in the mitochondria. Therefore, nuclear-encoded tRNA that is imported into the mitochondria must also be present in substantial abundances in the cytosol or elsewhere in the cell. The most notable exception was a low-abundance nuclear-encoded tRNA in *S. vulgaris* with homology to *alaCGC*. This tRNA was part of a family of related genes (supplementary table 1, Supplementary Material online, Reference ID#s 964, 1005–1014) with multiple sequence variants (supplementary fig. 4, Supplementary Material online) and reached comparable enrichment levels to those observed for mitochondrial genes (fig. 2). The anticodon of these tRNAs is uncertain, as an insertion in the anticodon loop may change the anticodon sequence from CGC to ACG (tRNA-Arg[ACG]) or possibly GCA (tRNA-Cys[GCA]), depending on how the tRNA folds in vivo (supplementary fig. 4, Supplementary Material online). Multiple tRNAs originating from these genes were found to be enriched in mitochondrial isolates, but detection was low and only one (nuclear ID: 1009, supplementary table 1, Supplementary Material online) survived the minimum read count threshold for differential expression analysis (fig. 2 and supplementary table 8, Supplementary Material online). Both tRNA-Ala(GCC) and tRNA-Arg(ACG) are expected to be imported into all angiosperm mitochondria (Michaud et al. 2011), and tRNA-Cys(GCA) is expected to be imported into *S. vulgaris* because of the recent loss of the mt-*cysGCA* gene, but why enrichment of these noncanonical tRNAs would be so high in mitochondrial isolates is unclear. It is possible that this is not a case of exclusive import but instead a failure of quality control, as import may have occurred prior to degradation, and the cytosolic surveillance mechanisms that degrade tRNAs without proper structures (Dewe et al. 2012) may not be active in the mitochondrial matrix. Other explanations for this enrichment are possible, but regardless these genes were the exception, as there was a clear distinction in the enrichment of mt-tRNAs versus the nuclear-encoded tRNA populations (fig. 2).

### Confirming the Utility of tRNA-Seq Methods to Detect the Import of Nuclear-Encoded tRNAs by Sequencing Mitochondrial tRNA Pools in *Solanum tuberosum*

Because *S. tuberosum* (potato) has had the most extensive prior characterization of mitochondrial tRNA import using northern blot analysis (Marechal-Drouard et al. 1990; Kumar et al. 1996; Brubacher-Kauffmann et al. 1999; Delage et al. 2003; Pujol et al. 2008), we sequenced the mitochondrial tRNA pools in *S. tuberosum* in order to compare the effectiveness of detecting tRNA import using the tRNA-seq methods employed in this study. Mitochondria were isolated from young *S. tuberosum* leaves with the same protocol and Percoll gradients used for *Sileneae* species.

Enrichment of *S. tuberosum* mitochondria in the isolated fractions was apparent from the high (21-fold-change) relative abundance of mt-tRNA and mitochondrial stem-loop sequences compared to the total-cellular samples (supplementary fig. 5 and table 6, Supplementary Material online). Likewise, plastid tRNAs were greatly depleted relative to mitochondrial tRNAs, indicating that the preparation of the mitochondrial isolates was also effective (although not perfect) at removing plastid tRNAs (table 2). As there is no known import of plastid tRNAs into mitochondria, any detection of plastid tRNAs in mitochondrial fractions likely represents contaminating plastids in the mitochondrial gradient purification.

Previous experiments testing for the exclusion of cytosolic tRNAs from *S. tuberosum* mitochondria (Marechal-Drouard et al. 1990; Brubacher-Kauffmann et al. 1999) reported a strong exclusion of tRNAs with the same anticodon as those that are still encoded in the mitogenome. In order to compare the sequence data generated here to previously performed hybridization methods (which detects the abundance of multiple tRNAs with the same anticodon), we calculated an average mitochondrial enrichment (log_2_ fold-change) weighted by expression of all tRNA sequences sharing the same anticodon (supplementary table 10, Supplementary Material online). The resulting mitochondrial enrichment values for tRNAs grouped by anticodon were highly consistent with previous northern blot results on enrichment and depletion of nuclear-encoded tRNAs in the mitochondrial fraction (supplementary table 11, Supplementary Material online). The seven tRNAs previously shown to be imported almost universally had higher enrichment values than the 17 tRNAs previously shown to be excluded—the only exception being that that tRNA-Gln(UUG) (excluded) had a slightly higher enrichment value than tRNA-Thr(UGU) (imported) (supplementary table 11, Supplementary Material online). The support for expected import and exclusion patterns for cytosolic tRNAs in *S. tuberosum* included the clear differential import of tRNA-Gly(CCC) and tRNA-Gly(UCC) transcripts but exclusion of tRNA-Gly(GCC) (supplementary table 11, Supplementary Material online) (Brubacher-Kauffmann et al. 1999).

**Fig. 3.**
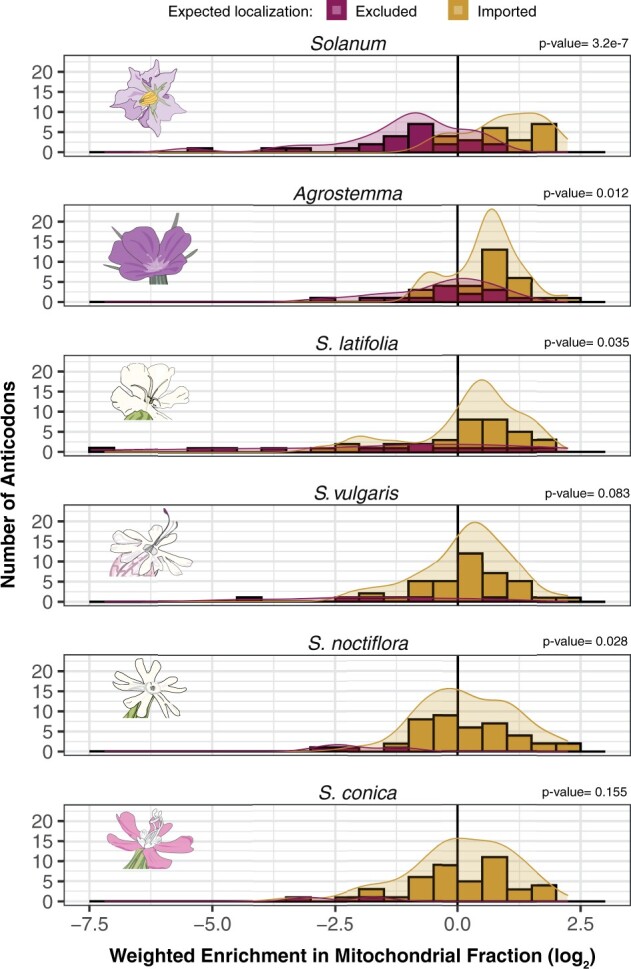
Histograms and density curves showing the distribution of average mitochondrial enrichments of nuclear-encoded tRNA anticodon families in *S. tuberosum, A. githago* and four *Silene* species. tRNAs with anticodons that are expected to be imported due to the lack of a corresponding mt-tRNA gene are shown in gold. Those that are expected to be excluded are shown in magenta. Reported *P*-values are based on *t*-tests between these two groups. Enrichment values are calculated based on expression-weighted average of all tRNAs with the same anticodon (see Materials and Methods). Positive and negative values indicate enrichment and depletion in the mitochondrial fraction, respectively.

Because only a subset of *S. tuberosum* tRNAs have been previously assayed for mitochondrial import by northern blot, we extended our analysis of the tRNA-seq data by predicting import/exclusion status for the remaining anticodons. An isodecoder family was predicted to be excluded if a tRNA for that anticodon was still encoded in the *S. tuberosum* mitogenome or imported if there was no corresponding tRNA encoded in the mitogenome. Correspondence between tRNA-seq enrichment/depletion and expected import status was highly significant (i.e., tRNAs that were predicted to be imported were significantly more enriched in the mitochondrial fraction than those expected to be excluded; Welch’s *t*-test, *P*-value = 3.24e−07, fig. 3).

### Extensive Shifts in Mitochondrial Import and Exclusion of tRNAs in *Sileneae* with Possible Cases of Redundant tRNA Import

We wanted to test whether rapid mt-tRNA gene loss in *Sileneae* precipitated shifts in cytosolic tRNA trafficking through changes in the import and exclusion of certain tRNA families. Like in *Solanum*, a signal of tRNA exclusion and import was also recovered for *Sileneae* species, with tRNA exclusion corresponding to some anticodons retained in the mitogenomes (i.e., a signal of nonredundant import of nuclear-encoded tRNAs). For all five *Sileneae* species, most cytosolic tRNAs predicted to be excluded from mitochondria because they share an anticodon with a mitochondrially encoded tRNA showed lower relative abundance on average than those predicted to be imported (fig. 3). This difference was not statistically significant for *S. conica* and *S. vulgaris* (fig. 3), but that may simply reflect the limited statistical power in species where so few tRNAs are predicted to be excluded because of extensive tRNA gene loss from the mitogenome. Even in these species, the cytosolic tRNAs that corresponded to the few remaining anticodons remaining in the mitogenomes were often the most depleted tRNAs (supplementary tables 3–8 and 10, Supplementary Material online). For example, cytosolic tRNA-Ile(UAU) was always one of the most strongly excluded tRNA families in all species, and mt-*ileCAU* is the only mt-tRNA gene retained in all species tested in this analysis (fig. 1, although the anticodon of mt-*ileCAU* would typically decode the Met codon AUG, the wobble position of the mt-tRNA is modified to a lysidine changing the specificity to the Ile codon AUA [Soma et al. 2003]).

**Fig. 4.**
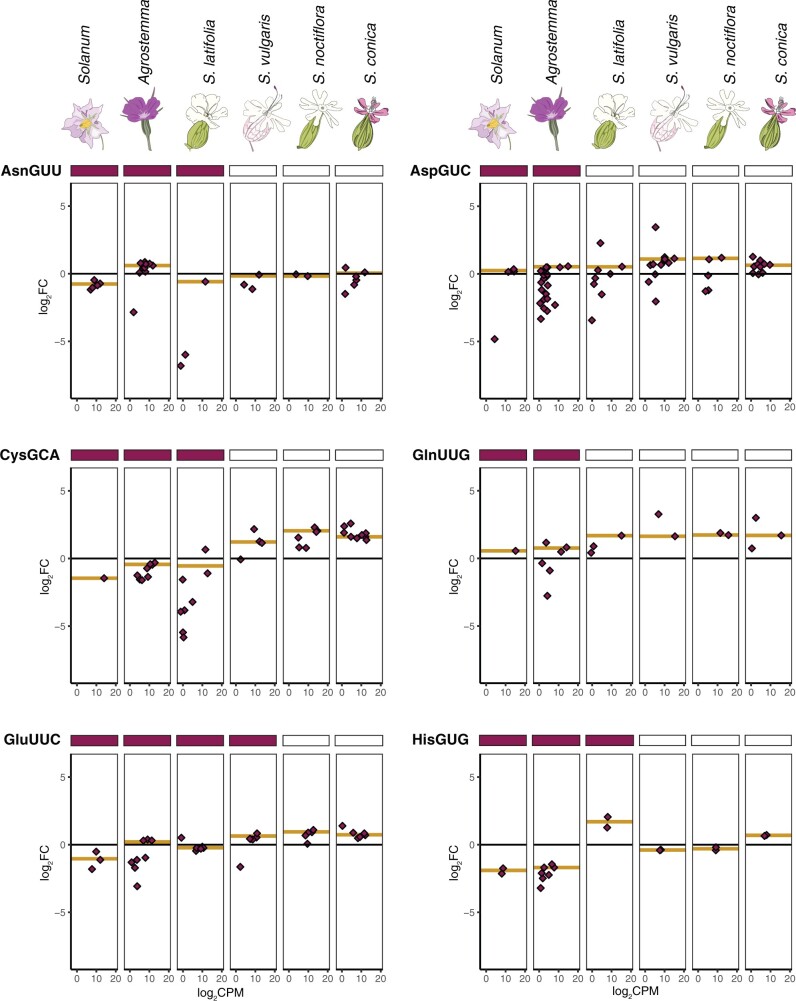
Enrichment and expression of individual cytosolic tRNA genes corresponding to mitochondrial genes with a history of loss in *Silene* since divergence from *Agrostemma* (first half, see fig. 5 for second half). The *y*-axis is enrichment in log_2_ fold change in mitochondrial isolates versus total-cellular samples. The *x*-axis is expression level in counts per million on a log_2_ scale. Points represent individual reference sequences, and gold lines represent the average enrichment of all nuclear-encoded tRNAs with that anticodon weighted by expression (see Materials and Methods; enrichment data for all anticodons can be found in supplementary table 10, Supplementary Material online). A filled rectangle above a panel indicates that a tRNA gene with that anticodon is encoded in the mitogenome of that species.

**Fig. 5.**
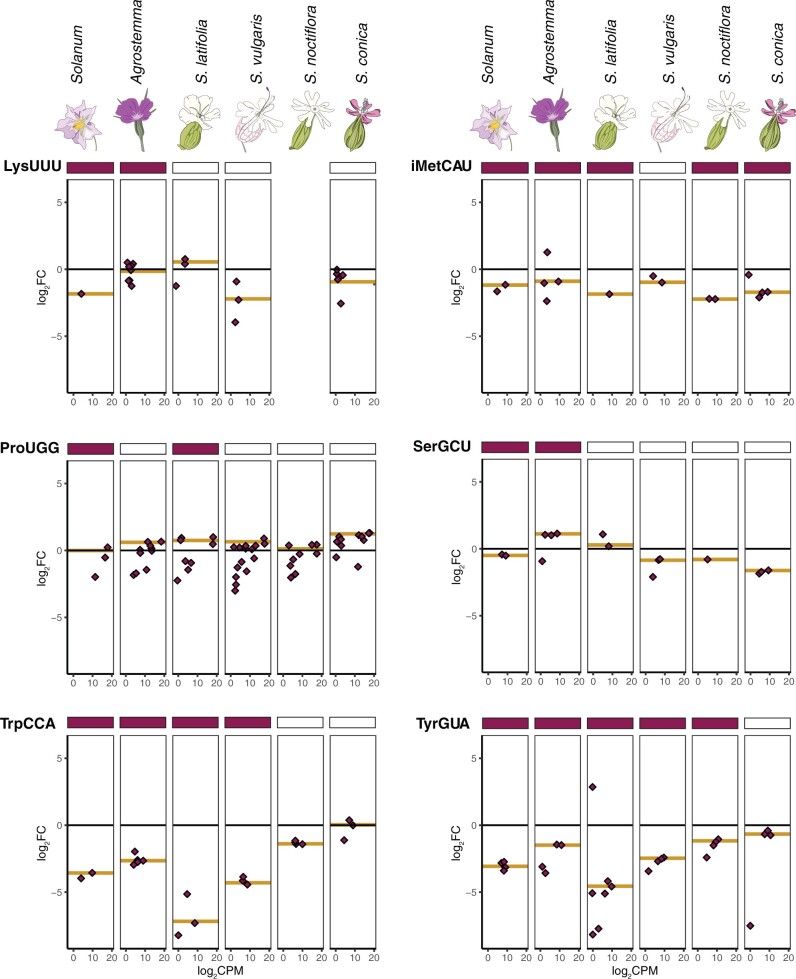
Enrichment and expression of individual cytosolic tRNA genes corresponding to mitochondrial genes with a history of loss in *Silene* since divergence from *Agrostemma* (second half, see fig. 4 for first half). No panel is reported for *S. noctiflora* tRNA-Lys because no tRNA-Lys gene was detected at sufficient expression levels of analysis of differential expression in that species. Cytosolic initiator methionine is abbreviated iMetCAU.

We predicted that *Sileneae* species that had lost a mt-tRNA gene would exhibit evidence of increased import of the corresponding nuclear-encoded tRNA from the cytosol. Overall, of the 12 mt-tRNA genes lost in at least one *Silene* species since divergence from *Agrostemma*, seven corresponding cytosolic tRNAs (tRNA-Asp[GUC], tRNA-Cys[GCA], tRNA-Gln[UUG], tRNA-Glu[UUC], tRNA-His[GUG], tRNA-Trp[CCA] and tRNA-Tyr[GUA]) showed increased representation in the mitochondrial fraction in all species that had lost the mt-tRNA counterpart compared to both *Solanum* and *Agrostemma* (figs. 4 and 5). Conversely, there were no cases showing the opposite pattern, where all *Silene* species with an mt-tRNA gene loss had lower enrichment than both *Solanum* and *Agrostemma* for the corresponding cytosolic tRNA. In addition, there are two mt-tRNA genes that were lost in the *Sileneae* lineage prior to the divergence of *Agrostemma* and *Silene*: tRNA-Gly(GCC) and tRNA-Met(CAU) (fig. 1). In both of these cases, the corresponding cytosolic isodecoder families showed higher mitochondrial enrichment in all five *Sileneae* species than *Solanum* (supplementary fig. 6, Supplementary Material online). In contrast to these tRNAs with a recent history of loss of the corresponding mt-tRNA genes in *Silene*, the nuclear-encoded tRNAs that are expected to be universally imported in angiosperms because of more ancient losses from the mitogenome did not show consistent trends toward mitochondrial enrichment in *Silene* relative to *Agrostemma* and *Solanum* (supplementary fig. 6, Supplementary Material online).

In some cases of mt-tRNA gene loss, we did not see expected changes in import. For example, the cytosolic initiator tRNA-Met(CAU) did not exhibit positive enrichment in any species (fig. 5), including *S. vulgaris* which lacks an intact copy of the functionally equivalent *f-metCAU* from its mitogenome—possibly suggesting that the cytosolic initiator tRNA-Met(CAU) may not be used for mitochondrial translation initiation even in systems that lose tRNA-fMet(CAU). Multiple tRNA-Ser anticodons failed to show a consistent pattern of increased enrichment despite multiple losses of tRNA-Ser genes in *Sileneae* mitogenomes but did exhibit substantial heterogeneity in enrichment across the different species (supplementary fig. 6, Supplementary Material online).

Interestingly, there were multiple cases of increased mitochondrial enrichment of a cytosolic tRNA in a *Sileneae* species that still retained the corresponding mt-tRNA gene. Such cases suggest that there may be sustained periods of time where mitochondrially encoded tRNAs and imported cytosolic tRNAs with the same anticodon exist redundantly within mitochondria. For tRNA-His(GUG) in *S. latifolia*, tRNA-Glu(UUC) in *S. vulgaris*, and tRNA-Asn(GUU) in *A. githago* (figs. 4 and 5 and supplementary fig. 6, Supplementary Material online), there was a positive enrichment value despite the species retaining a mitochondrial counterpart in the mitogenome.

## Discussion

The import of tRNAs across mitochondrial membranes occurs in various eukaryotic lineages (Salinas-Giegé et al. 2015), but questions remain about the evolutionary mechanisms that facilitate the functional replacement and integration of nuclear-encoded tRNAs in mitochondrial translation.

Except for a handful of tRNA-Ala(CGC) paralogs in *S. vulgaris*, we did not find nuclear-encoded tRNAs enriched to the same level as those encoded in the mitogenome (fig. 2). This result suggests an absence of dedicated tRNAs for mitochondrial import in these systems with recent mitochondrial tRNA gene replacement. It is yet to be determined if these imported tRNAs are aminoacylated, but the widespread sharing of nuclear-encoded tRNAs between both compartments is notably different from the imported mitochondrial proteome, which has been estimated to only be 15% dual-localized between the mitochondria and another cellular location (Calvo and Mootha 2010). This lack of tRNA specialization contrasts with mitochondrial tRNA import studies performed in the green alga *Chlamydomonas* in which multiple nuclear-encoded tRNAs had varying degrees of mitochondrial specialization, and a nuclear-encoded tRNA-Lys(UUU) was found to be exclusively localized to the mitochondria (Vinogradova et al. 2009).

One caveat to this interpretation is that, even if tRNAs are destined for exclusive function in the mitochondria, there may be a substantial fraction “en route” at any given point in time. Therefore, they would still be present in substantial quantities elsewhere in the cell, producing a muted mitochondrial enrichment signal. This caveat aside, the dual localization of tRNAs between the cytosol and mitochondria represents a large-scale homogenization of host (nuclear) and endosymbiont (mitochondrial) translational systems.

Sequencing tRNA pools from isolated mitochondria of multiple *Sileneae* species revealed substantial changes in the relative abundance of nuclear-encoded tRNAs corresponding to mt-tRNA gene loss (figs. 4 and 5 and supplementary fig. 6, Supplementary Material online). These fluctuations occurred on phylogenetically recent timeframes, suggesting that changes to tRNA import specificity can occur rapidly. However, the *de novo* evolution of tRNA import did not appear to be a perfectly binary switch from excluded to imported. There were cases where a nuclear-encoded tRNA family showed partially elevated abundance in the mitochondrial fraction of a species that still retained a mitochondrially encoded counterpart (see tRNA-Glu[UUC] and tRNA-Tyr[GUA] in figs. 4 and 5). Loss of exclusion signals prior to mt-tRNA gene loss may point to a model where the evolution of tRNA import is a gradual process whereby some cytosolic “leakiness” is tolerated. If these imported tRNAs are functional, we hypothesize that leaky tRNA import may provide the necessary first step in tolerating mt-tRNA gene losses by providing functional redundancy.

And finally, it is interesting to note that some of the mt-tRNA genes most recalcitrant to loss in *Silene* (*ileCAU*, *trpCCA*, and *tyrGUA*) corresponded to the most excluded cytosolic tRNAs in *Solanum*. It may be that certain tRNAs interact with additional/different enzymatic partners during cellular trafficking, thereby preventing the indiscriminate import and resulting in strong exclusion signals. The successful delivery of these tRNAs to functionally sufficient levels would then require additional coevolutionary hurdles to overcome before functional replacement.tRNAs are not standalone molecules in the process of translation, as entire networks of enzymes must interact with tRNAs for maturation and function. Because some tRNA-interacting enzymes are highly specific (Chimnaronk et al. 2005; Salinas-Giegé et al. 2015), separate sets of enzymes are normally required for the maturation and aminoacylation of cytosolic vs. organellar tRNAs (Duchêne et al. 2009). How nuclear-encoded tRNAs are effectively “swapped” into the mitochondrial translational machinery has remained a long-standing question in plant mitochondrial genetics (Warren and Sloan 2020). Notably, the charging of tRNAs with the correct amino acid is necessary for protein synthesis and relies on aaRSs, which use specific nucleotide identities along with the tRNA for recognition and interaction with cognate tRNAs (Giegé et al. 1998; Meiklejohn et al. 2013). Without an effective aaRS partner, a newly imported tRNA would be nonfunctional in translation. One possible evolutionary mechanism would involve a cytosolic aaRS also gaining mitochondrial import and charging the cognate, imported tRNA present in the mitochondrial matrix. Massive tRNA loss in the mitogenomes of some nonbilaterian animals (e.g., sponges) has been associated with the loss of mitochondrial aaRSs, suggesting that imported tRNAs are being charged by partnered cytosolic aaRSs (i.e., replacement of both tRNA and aaRS) (Haen et al. 2010; Pett and Lavrov 2015). However, this presents what has been previously termed the “chicken and egg” problem in plant mt-tRNA replacement (Small et al. 1999). Does the aaRS or the tRNA gain import first if neither would be functional without the other? Our results from sequencing of tRNAs from the mitochondrial fractions of *Sileneae* species as well as *S. tuberosum* may provide some insight into this conundrum—cytosolic tRNAs not essential for mitochondrial translation (because of the presence and expression of a native mt-tRNA counterpart) may frequently be found in the mitochondrial matrix before the loss of mt-tRNA genes. It is unknown if these imported tRNAs are aminoacylated and functional, and if so, whether a mitochondrial aaRSs has evolved increased substrate recognition to charge both mitochondrial- and nuclear-encoded tRNAs, or if cytosolic aaRSs has also gained mitochondrial import alongside the cognate tRNA.

The homogenization of tRNA metabolism in plant mitochondria represents a much different evolutionary route than that taken by most bilaterian animals, which have retained an independent and conserved set of mt-tRNAs with very limited tRNA import into mitochondria (Grosjean and Westhof 2016). Fundamental differences in the historical mutation rates of mitogenomes in animals versus plants may have created a situation whereby plant mt-tRNAs are more predisposed to the functional replacement with that of cytosolic counterparts. The rate of mitochondrial sequence evolution in land plants is among some of the slowest ever estimated (Mower et al. 2007) and has likely been a factor in the maintenance of the largely canonical shape of plant mt-tRNAs. This conserved shape may contribute to the interchangeable nature of plant mt-tRNA genes. It is possible that the slow rate of evolution in plant mitogenomes has resulted in tRNA enzymatic networks that are more amenable to nuclear-encoded substrates because of shared sequence identity and structure between plant mt-tRNAs and nuclear counterparts. In contrast, the much faster-evolving animal mitogenomes (Brown et al. 1979; Lavrov and Pett 2016) may have experienced early tRNA mutations and compensatory enzymatic substitutions that make nuclear-encoded tRNAs poor substrates for the tRNA-interacting enzymes that function in the mitochondria (Watanabe et al. 2014; Pons et al. 2019)—thereby locking in the independence of the mitochondrial protein synthesis apparatus.

Despite this generally low mutation rate, some plant lineages have experienced a recent and dramatic acceleration in mutation rates in their mitogenomes, including multiple species in *Silene* (Mower et al. 2007; Sloan et al. 2009; Broz et al. 2021). This accelerated rate may have then facilitated a rapid replacement of mt-tRNA genes in some plant lineages. *Silene conica* and *S. noctiflora* both have highly accelerated rates of mitochondrial sequence evolution, as well as the fewest reported mt-tRNA genes in angiosperms, with only two and three mt-tRNA genes, respectively (fig. 1). It has been proposed that high mutation rates increase the likelihood of disruptive mutations in tRNAs and provide a selective pressure to functionally replace mt-tRNA genes with imported nuclear-encoded counterparts—thereby ratcheting a gene replacement process (Brandvain and Wade 2009). Another plant lineage with a high rate of sequence evolution, *Viscum* (mistletoe), also has a severely reduced mt-tRNA gene content (Petersen et al. 2015; Skippington et al. 2015). The heterogeneity in mitogenomes across angiosperm diversity creates exciting opportunities to test these hypothesized relationships between import specificity, mutation rates, and the functional loss/replacement of mt-tRNAs.

## Materials and Methods

### *Agrostemma githago* and *S. chalcedonica* Mitochondrial Genome Sequencing

Mitochondrial DNA was extracted from *A. githago* and *S. chalcedonica* using methods described previously (Sloan et al. 2010; Sloan, Alverson, Wu, et al. 2012). Tissue was collected from the same maternal families previously used for plastid genome sequencing in these species (Sloan et al. 2014). Mitochondrial DNA from each species was used to construct 3 kb paired-end libraries which were sequenced on a Roche 454 GS-FLX platform with Titanium reagents. Library construction and sequencing were performed at the University of Virginia’s Genomics Core Facility. Reads were assembled with Roche’s GS de novo Assembler v2.3 (“Newbler”) using default settings, resulting in average read depths of >50× for the mitogenomes of each species. The mitogenome of *A. githago* was then manually assembled into a master circle confirmation based on the Newbler assembly graph as described previously (Sloan, Alverson, Wu, et al. 2012). We did not attempt to manually close the assembly of the larger and more complex *S. chalcedonica* mitogenome. In order to correct the known high insertion and deletion error rates associated with long homopolymer regions from 454 sequencings, we performed Illumina sequencing on total-cellular DNA from an individual from the same *A. githago* maternal family (Giles County) used for mitogenome sequencing. DNA was extracted from a 3-day old seedling using a CTAB protocol (Doyle and Doyle 1987). An Illumina library was constructed with the NEBNext Ultra II FS DNA Library Prep Kit, using approximately 30 ng of input DNA, a 20 min fragmentation time, and 8 cycles of PCR amplification. The library was dual-indexed and multiplexed with other libraries on a NovaSeq 6000 S4 sequencing lane (paired-end, 150-bp reads) at the University of Colorado Cancer Center, generating 49.6M read pairs. These reads were then mapped to the mitogenome to correct homopolymer length errors in an iterative fashion as described previously (Sloan, Alverson, Wu, et al. 2012).

The *A. githago* mitogenome was annotated with Mitofy (Alverson et al. 2010), BLAST homology searches, and tRNAscan-SE (Chan and Lowe 2019). tRNAscan-SE was also used to identify tRNA genes in the mitochondrial contigs of the *S. chalcedonica* assembly. The mitogenome plot (supplementary fig. 1, Supplementary Material online) was produced with OGDRAW version 1.3.1 (Greiner et al. 2019). The annotated *A. githago* mitogenome sequence and the contigs from the *S. chalcedonica* assembly were deposited to GenBank (accession numbers MW553037 and MW580967-MW581000, respectively). The raw 454 and Illumina reads are available via NCBI SRA (accessions SRX9983705, SRX9983703, and SRX9983702).

### Tissue Growth for tRNA Analysis

*Agrostemma githago* seeds were obtained from the Kew Gardens Millennium Seed Bank (Kew 0053084), germinated in small plastic pots with Pro-Mix BX General Purpose soil supplemented with vermiculite and perlite, and grown in growth chambers held at 23 °C with a 16-h/8-h light/dark cycle (light intensity of 100 µE⋅m^−2^⋅s^−1^). *Silene latifolia* seeds were from a female derived from the line originally used for mitogenome sequencing (Sloan et al. 2010) fertilized by a male obtained from the Kew Gardens Millennium Seed Bank (Kew 32982). Leaf tissue for *S. tuberosum* was generated by planting seed potato (tubers) cultivar White Kennebec. *Silene latifolia*, *S. noctiflora* BRP (Wu et al. 2015), and *S. vulgaris* S9L (Sloan, Muller, et al. 2012) seeds were germinated in SC7 Containers (Stuewe and Sons) with the same soil as above on a mist bench under supplemental lighting (16-h/8-h light/dark cycle) in the Colorado State University greenhouse. After germination, *S. latifolia*, *S. noctiflora*, and *S. vulgaris* seedlings were moved to a bench with the same 16-h/8-h light/dark cycle supplemental lighting until harvest. *S. conica* ABR (Sloan, Alverson, Chuckalovcak, et al. 2012) seeds were germinated in the same soil and containers but grown in a growth room under short-day conditions (10-h/14-h light/dark cycle) in order to increase rosette growth. And finally, *S. tuberosum* seed potato was planted in the same soil but in one-gallon pots in the same chamber and short-day conditions (10-h/14-h light/dark cycle) to promote leaf tissue growth.

### Mitochondrial Isolations

The age of the plants at harvest time ranged depending on species, as certain species required more growth time to produce a sufficient amount of tissue for mitochondrial isolations. *Agrostemma githago* plants were harvested at 4 weeks, *S. conica* at 14 weeks*, S. latifolia* at 6 weeks, *S. noctiflora* at 14 weeks, *S. vulgaris* at 8 months, and *S. tuberosum* at 4 weeks. Seventy-five grams of leaf and stem tissue (entire above ground tissue) was collected for each replicate for each respective species. This represented 5 potato plants and anywhere from 24 to 65 *Sileneae* plants per replicate. Leaf tissue was disrupted in a Nutri Ninja Blender for 2 × 2-s short bursts, and 1 × 4-s blending in 350 ml of a homogenization buffer containing: 0.3 M sucrose, 5 mM tetrasodium pyrophosphate, 2 mM EDTA, 10 mM KH_2_PO_4_, 1% PVP-40, 1% BSA, 20 mM ascorbic acid, and 5 mM cysteine (pH 7.5-KOH) in a cold room. Homogenate was then poured over four layers of cheesecloth and 1 layer of Mira cloth and squeezed to filter out all solids. The liquid homogenate was then subjected to differential centrifugation to remove nuclei, plastids, and cellular debris in a Beckman-Coulter Avanti JXN-30 centrifuge with a JA14.50 fixed-angle rotor at 4 °C. Homogenate was spun at 500 rcf, 1,500 rcf, and 3,000 rcf for 10 min at each speed. After each centrifugation step, the supernatant was transferred into a clean centrifuge bottle. Mitochondria were then pelleted by centrifugation for 10 min at 20,000 rcf with the brake off. The supernatant was discarded and the pellet was resuspended using a goat-hair paintbrush and 2 ml wash buffer containing 0.3 M sucrose, 10 mM MOPS, 1 mM EGTA (pH 7.2-KOH). The supernatant was then transferred to 32 ml tubes for centrifugation on a swinging bucket rotor (JS-24.38). The homogenate was centrifuged for 5 min at 3,000 rcf to pellet residual plastid and nuclear contaminants, and the supernatant was transferred to a clean 32 ml tube. Mitochondria were once again pelleted by centrifugation for 10 min at 20,000 rcf. The pellet was resuspended with 500 µl wash buffer and a paintbrush. The mitochondrial suspension was then added to a glass Dounce homogenizer and homogenized with three strokes.

Homogenized mitochondria were then suspended on top of a Percoll gradient with the following Percoll density layers, 18%, 25%, and 50%. The gradient was then centrifuged at 40,000 rcf for 45 min with the brake off. The mitochondrial band at the 25:50% interface was then aspirated off of the gradient and diluted with 30 ml of wash buffer. The diluted mitochondria were then centrifuged at 20,000 rcf for 10 min. The supernatant was vacuum aspirated, and the mitochondrial pellet was resuspended in a fresh 30 ml of wash buffer and centrifuged at 10,000 rcf for 10 min. The supernatant was vacuum aspirated, and the mitochondrial pellet was resuspended in 1,000 µl of fresh wash buffer. Resuspended mitochondria were centrifuged at 10,000 rcf for 10 min. The supernatant was removed with a pipette and the mitochondrial pellet immediately went into RNA extraction procedures using 1,000 µl TRIzol following the manufacturer’s RNA extraction protocol.

The total-cellular samples for *A. githago, S. latifolia, and S. noctiflora* were produced by freezing an entire, single plant excluding any root tissue in liquid nitrogen and grinding with a mortar and pestle into powder and performing an RNA extraction using the Trizol manufacturer’s protocol. In the case of *S. tuberosum*, *S. vulgaris*, and *S. conica*, one shoot from one plant with all leaves was used.

Only two replicates of *S. latifolia* were performed because of tissue limitations, and only two mitochondrial isolations from *S. vulgaris* were used in the downstream analysis because of an error quantifying the AlkB/RNA ratio in one of the isolations. Two replicates of mitochondrial isolations and total cellular samples were produced for *S. tuberosum.* Three replicates (mitochondrial isolations and total-cellular samples) were produced for all other species.

### AlkB Treatment and YAMAT-Seq Library Construction

See Warren et al. (2021) for detailed protocols for AlkB expression/purification, RNA treatment, and YAMAT-seq (tRNA-seq) library construction. Briefly, a plasmid containing cloned wild-type AlkB protein (pET24a-AlkB deltaN11 [plasmid #73622]) was obtained from Addgene (http://www.addgene.org/), and the AlkB protein was expressed and purified at the CSU Biochemistry and Molecular Biology Protein Expression and Purification Facility.

For the demethylation (AlkB) reaction, 6 µg of total RNA was treated with 250 pmols of AlkB for 1 h at 37 °C in a reaction buffer containing 70 µM ammonium iron(II) sulfate hexahydrate, 0.93 mM α-ketoglutaric acid disodium salt dihydrate, 1.86 mM ascorbic acid, and 46.5 mM HEPES (pH 8.0-HCl). The reaction was quenched by adding 4 µl of 100 mM EDTA. RNA was then extracted with a phenol-chloroform RNA extraction, followed by ethanol precipitation with the addition of 0.08 µg of RNase-free glycogen and resuspension in RNase-free water. RNA integrity was checked on a TapeStation 2200.

To remove amino acids attached to the 3′-end of tRNAs prior to adapter ligation, the demethylated RNA was deacylated in 20 mM Tris HCl (pH 9.0) at 37 °C for 40 min. Following deacylation, adapter ligation was performed using a modified protocol from Shigematsu et al. (2017). A 9 µl reaction volume containing 1 µg of deacylated RNA and 1 pmol of each Y-5′ adapter (4 pmols total) and 4 pmols of the Y-3′ adapter was incubated at 90 °C for 2 min. 1 µl of an annealing buffer containing 500 mM Tris-HCl (pH 8.0) and 100 mM MgCl_2_ was added to the reaction mixture and incubated for 15 min at 37 °C. Ligation was performed by adding 1 unit of T4 RNA Ligase 2 enzyme (New England Biolabs) in 10 µl of 1X reaction buffer and incubating the reaction at 37 °C for 60 min, followed by overnight incubation at 4 °C. Reverse transcription of ligated RNA was performed using SuperScript IV (Invitrogen) according to the manufacturer’s protocol.

The resulting cDNA was then amplified by PCR using NEBNext PCR Master mix with 7 µl of the reverse transcription reaction. Ten cycles of PCR were performed for *A. githago*, *S. noctiflora*, and *S. vulgaris*. To generate greater library yields, we increased the number of PCR cycles to 12 cycles for *S. conica*, *S. latifolia*, and *S. tuberosum.* PCR was performed on a Bio-Rad C1000 Touch thermal cycler with an initial 1 min incubation at 98 °C and 10/12 cycles of 30 s at 98 °C, 30 s at 60 °C and 30 s at 72 °C, followed by 5 min at 72 °C. Size selection of the resulting PCR products was done on a BluePippin (Sage Science) with Q3 3% agarose gel cassettes following the manufacturer’s protocol. The size selection parameters were set to a range of 180–231 bp. Size-selected products were then cleaned using solid-phase reversable immobilization (SPRI) beads and resuspended in 10 mM Tris (pH 8.0). Libraries were dual-indexed and sequenced on an Illumina NovaSeq 6000 S4 lane with paired-end, 150-bp reads at the University of Colorado Cancer Center. YAMAT-seq reads for *A. githago*, *S. conica*, *S. latifolia*, *S. noctiflora* and *S. tuberosum* are available via the NCBI Sequence Read Archive under BioProject PRJNA698234 and sequencing reads for *S. vulgaris* are available under BioProject PRJNA662108.

### YAMAT-Seq Read Processing

Adapters were trimmed using Cutadapt version 1.16 (Martin 2011) with a nextseq trim quality-cutoff parameter of 20 (option: –nextseq-trim = 20). A minimum length filter of 50 bp and a maximum of 95 bp was applied to only retain full-length tRNA sequences and remove a substantial number of reads containing only adapter sequence without an insert (Shigematsu et al. 2017). BBMerge from the BBTools software package was used to merge R1 and R2 read pairs into a consensus sequence (Bushnell et al. 2017). Identical reads were summed and collapsed into read families using the FASTQ/A Collapser tool from the FASTX-Toolkit version 0.0.13 (http://hannonlab.cshl.edu/fastx_toolkit/index.html).

### Nuclear Genome Assemblies and Reference tRNA Genes

In order to generate reference tRNA gene sequences for each species, nuclear genome assemblies were either generated from Illumina short-read data sets or obtained from published sources. Illumina reads for an *A. githago* nuclear assembly were generated from total-cellular DNA extracted from a 5-month-old plant germinated from a line started from Kew 0053084 seeds. DNA was extracted using a Qiagen DNeasy kit, and the NEBNext Ultra II FS DNA Library Prep Kit was used as described above. Sequencing was performed on an Illumina NovaSeq 6000 S4 Lane with paired-end 150-bp reads at the University of Colorado Cancer Center. Shotgun sequencing reads for *A. githago* are available via the NCBI Sequence Read Archive under BioProject PRJNA698248. Illumina reads for an *S. vulgaris* were generated from plants were grown from seeds collected from natural populations in Seefield, Austria. Leaf tissue was collected from a single hermaphrodite plant that had been crossed to the F2 generation and grown under standard greenhouse conditions at the University of Virginia. Genomic DNA was extracted with a modified CTAB protocol (Doyle and Doyle 1987) and sent to Global Biologics (Columbia, MO) for the construction of Illumina libraries with 180 bp inserts. Paired-end (2 × 100) reads were sequenced at the Yale Center for Genome Analysis on a single Illumina HiSeq 2000 lane. Raw reads have been deposited into the NCBI Short Read Archive (SRA) with accession SRX10073976. *Silene conica* ABR Illumina reads were obtained from a previously published data set (Broz et al. 2021).

Genomic reads for *A. githago*, *S. conica*, *S. vulgaris* were trimmed with cutadapt ver. 1.16 (Martin 2011) with a quality-cutoff parameter of 10 for the 3′ end of each read and a minimum length filter of 5 bp. Reads were then assembled using SPAdes v3.11.0, (Nurk et al. 2013) using the following command options: -k 21,33,55,77,99 -t 40 -m 900. Assemblies for *S. noctiflora* OPL (GenBank assembly accession: VHZZ00000000.1) and *S. latifolia* (GenBank assembly accession: QBIE00000000.1) were taken from previous studies (Krasovec et al. 2018; Williams et al. 2020).

A database of nuclear tRNA reference genes was produced by searching each of the nuclear assemblies from *A. githago*, *S. conica, S. latifolia*, *S. noctiflora*, and *S. vulgaris* with tRNAscan-SE. ver. 2.0.3 (Chan and Lowe 2019) using the General search option (-G). Introns predicted from tRNAscan-SE were removed, and identical tRNA sequences were collapsed into a single reference using custom Perl scripts. It is important to note that plant genomes have multiple copies of identical tRNA genes, but for mapping purposes, all of these identical sequences are collapsed into a single reference.

The nuclear references for *S. tuberosum* were obtained from the curated PlantRNA website (http://seve.ibmp.unistra.fr/plantrna/, downloaded October 26, 2020).

### Mitochondrial Genome Mapping and Stem-Loop References

To search for previously undetected mitochondrial tRNA sequences captured by YAMAT-seq, as well as to identify any expressed stem-loop regions modified with a CCA-tail, all YAMAT reads for each species were mapped to the entire mitogenome using BLAST (blastn, e-value of <1e−6, low complexity regions not filtered [-dust no]). To ensure only high confidence mitogenome hits reads mapping to the mitogenome were then filtered so that only reads with greater than or equal to 90% identical matches and hit coverage to a mitogenome location were retained. The “closest” function in bedtools ver. 2.27.1 (Quinlan and Hall 2010) was used to assign the closest annotated gene for each of these retained reads, including any reads that mapped/overlapped with a previously annotated tRNA. All reads that did not map to a tRNA gene were then further analyzed to determine if they occurred at the boundary of protein-coding or RNA gene (indicating that these transcripts may be t-elements). Those that did not occur at a gene boundary were considered to represent a previously undocumented stem-loop or tRNA-like structure. Highly expressed t-elements and stem-loops were added to the mapping database as reference sequences.

### Folding Predictions of Mitochondrial t-Elements and Stem-Loops

Folding prediction analysis for supplementary figure 2, Supplementary Material online was done with RNAFold (ver. 2.4.11 [Lorenz et al. 2011]), using the maximum free energy (MFE) model. Because stem-loops also had RT misincorporations at some sites, the YAMAT sequence with 100% nucleotide identity to the mitogenome was used for the folding prediction. Folding diagrams were created with VARNA (ver. 3.9 [Darty et al. 2009]).

### Contamination Removal

Although the vast majority of the YAMAT reads originated from the sampled plant species, there were numerous reads of contaminating origins such as soil bacteria and plant pests. In order to remove reads that did not originate from the plant itself, all unique sequences with three or more reads were BLASTED to the NCBI nucleotide collection (nt) database (posting date of October 27, 2019, downloaded February 02, 2020) (blastn, e-value of <1e−3), and the taxonomy information for the top two hits for each read was pulled using the NCBI taxonomy database (downloaded February 02, 2020). All queries that did not have the taxon name Viridiplantae in the first two hits were retained to make a contamination database. GenBank files were downloaded for all contaminating accessions that had more than 50 reads assigned, and tRNA annotations were extracted from those GenBank files. Some accessions did not have annotated tRNA genes, so the contaminating hit was added to the database manually. Many of the contaminating references were identical or nearly identical to each other (e.g., many bacterial tRNA sequences were identical or only differed by slight length variation due to annotations). Thus, the contaminant database was collapsed to retain only unique sequences and only the longest reference of otherwise identical sequences. Contaminating sequences were added to the mapping database as reference sequences. We also filtered YAMAT-seq reads that did not map to any reference with at least 90% sequence identity and 80% coverage (see below) to ensure that any low-abundance, contaminating reads were not retained in the analysis.

### Read Mapping

Mapping of processed reads was performed with a custom Perl script previously published in Warren et al. (2021) that can be found at (https://github.com/warrenjessica/YAMAT-scripts). Briefly, each read was BLASTed (blastn, e-value threshold of <1e−6) to all references. A mapped read was retained if it had 90% or more sequence identity and at least 80% coverage to a reference sequence. This ensured that full-length tRNA sequences were used in analysis but also allowed for some mismatches due to reverse transcription-induced misincorporations (Warren et al. 2021). Furthermore, only sequences that uniquely mapped to a single reference were retained for downstream analysis. All mapped and filtered reads were then counted for differential expression analysis.

### EdgeR Differential Expression Analysis

Differential expression analysis was done on mapped counts with the R package EdgeR version 3.24.3 (Robinson et al. 2010) using a gene-wise negative binomial generalized linear model with quasi-likelihood tests (glmQLFit). Library sizes were normalized with the function calcNormFactors(y) and low-abundance genes were filtered by expression using the filterByExpr(y) function. Because misincorporations in cDNA are common when reverse transcribing tRNAs, lowly detected (<2 counts per million or CPM) sequences in this analysis likely represent rare misincorporation profiles of tRNA sequences (and not truly unique genes) which occasionally mapped to unique references. To summarize the level of mitochondrial enrichment by the anticodon (isodecoder) family, a weighted average enrichment level for all tRNAs with the same anticodon was calculated and presented in figures 6 and 7. The average enrichment was calculated by weighting each sequence by its percentage of total expression (CPM) within the isodecoder family. Count data, as well as differential expression results, can be found in supplementary tables 2–8, Supplementary Material online, and the weighted enrichment for each anticodon group for each species can be found in supplementary table 11, Supplementary Material online.

## Supplementary Material

Supplementary data are available at *Molecular Biology and Evolution* online.

## Supplementary Material

msab255_Supplementary_Data
