# Supplementary material for: Rapid Shifts in Mitochondrial tRNA Import in a Plant Lineage with Extensive Mitochondrial tRNA Gene Loss
Source: Mol Biol Evol. 2021 Aug 26;38(12):5735–51. doi: 10.1093/molbev/msab255 (PMC8662596; doi:10.1093/molbev/msab255)
Supplement: msab255_Supplementary_Data [file msab255_supplementary_data.zip › Supp_figs_all_R1.pdf]

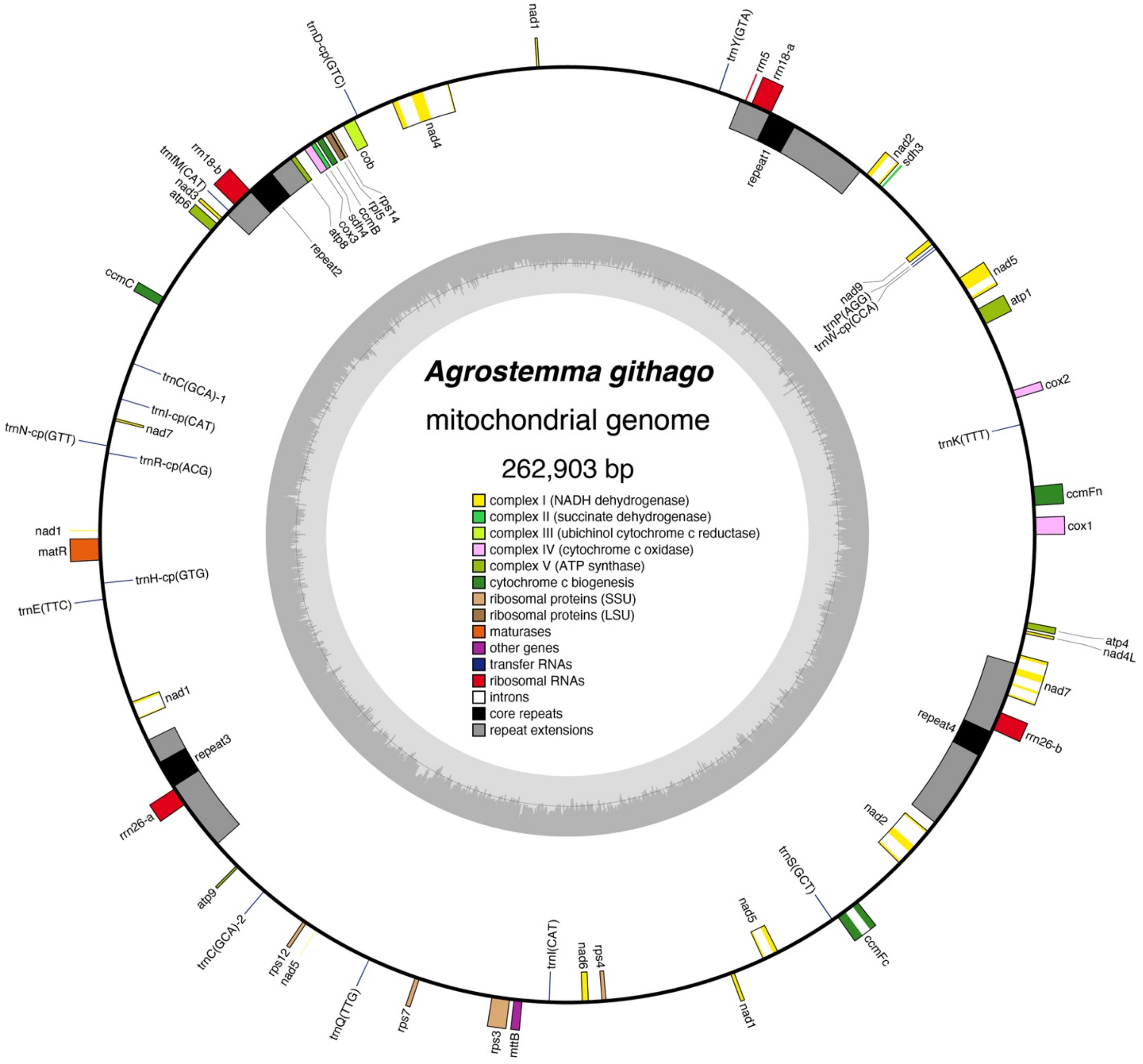

Supp. Fig.1| Mitogenome map of *Agrostemma githago*. This "master circle" represents only one possible configuration of the mitogenome due to multiple recombing repeats. Boxes inside and outside the circle correspond to the clockwise and anticlockwise strands, respectively. The black regions of the repeats designate the identical sequence shared by all four copies. The flanking gray boxes represent extensions to these repeat regions, which are present in two or more (but not all four) copies. The inner gray track represents GC content. Figure was generated with OGDraw v1.3.1 (Greiner, et al. 2019)

T-elements and stem-loops

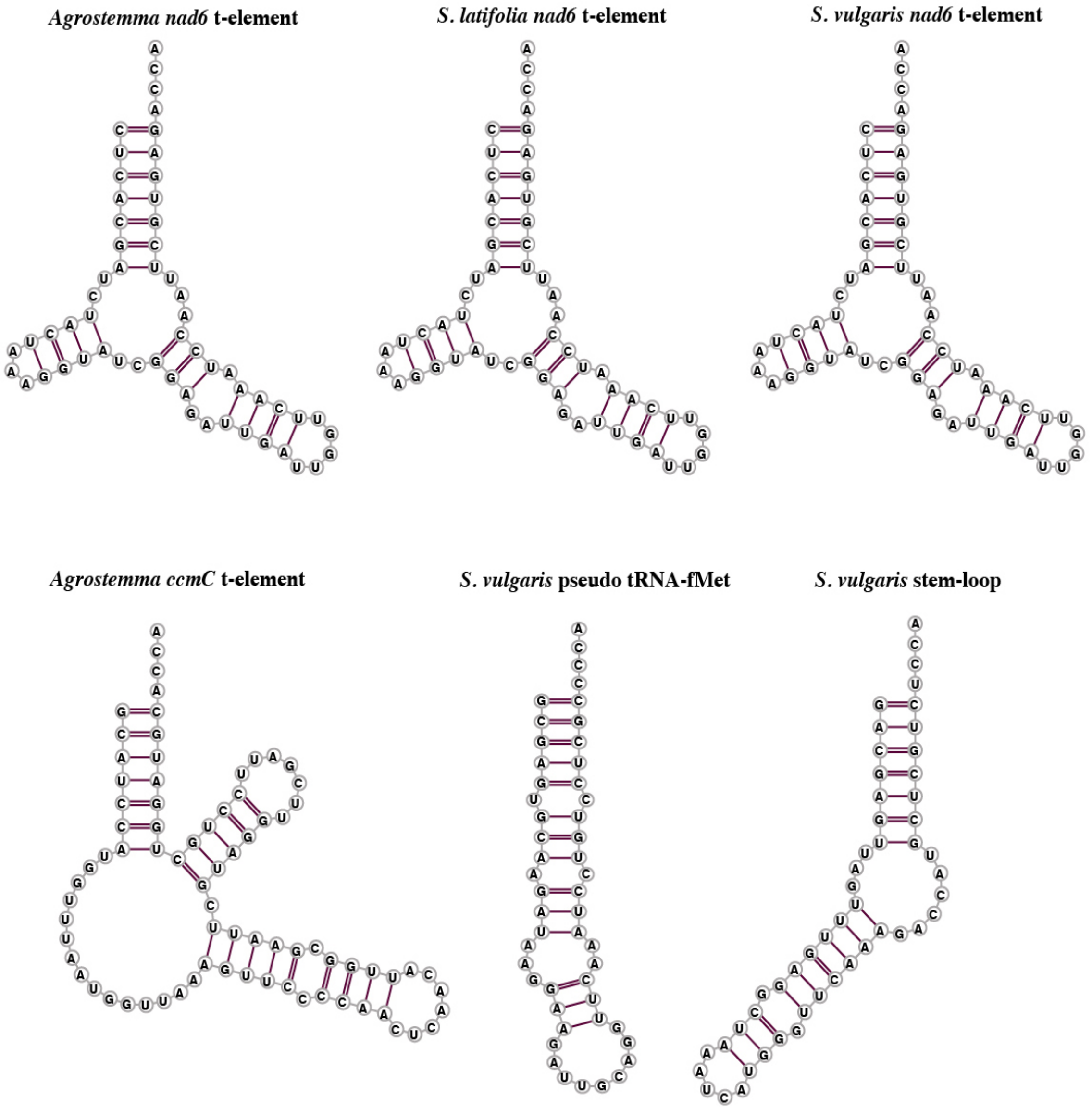

Supp. Fig. 2| Predicted foldings of some of the most highly detected stem-loop and t-element structures. Folding predictions were done using RNAfold (Lorenz, et al. 2011) with a maximum free energy model, and diagrams were created with VARNA ver. 3.9 (Darty, et al. 2009).

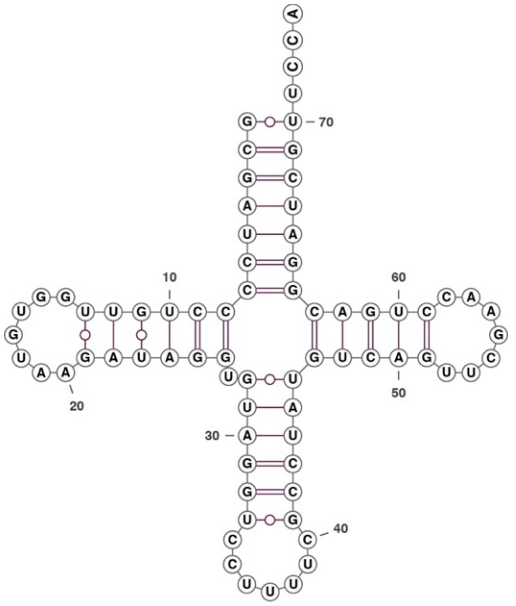

Supp. Fig. 3| Predicted cloverleaf folding transcript expressed and post-transcriptionally modified with a CCA-tail in *A. githago*. Despite the predicted tRNA-like secondary structure, this sequence is not homologous to any previously annotated mt-tRNA genes. Folding represents the maximum free energy folding model using the program the program RNAfold (Lorenz, et al. 2011), and structure diagrams were created using the program VARNA ver. 3.9 (Darty, et al. 2009).

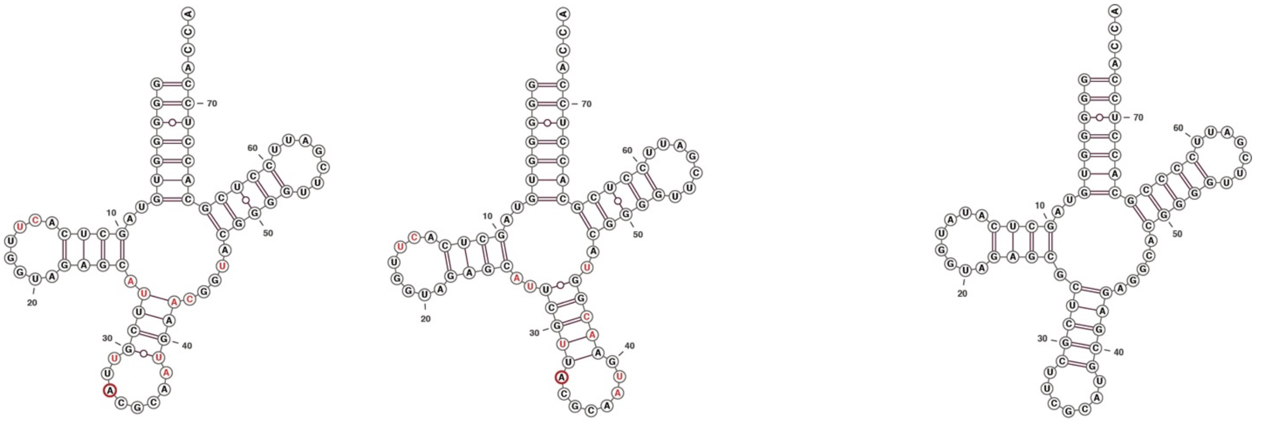

S. vulgaris nuclear gene (1009)

S. vulgaris nuclear gene (1009)
alternative folding

S. vulgaris AlaCGC (1015)

Supp. Fig. 4| Sequence and alternative predictive foldings of a highly enriched nuclear-encoded gene (#1009) in *S. vulgaris* mitochondrial isolates and the closest *S. vulgaris* reference with a tRNAscan-determined anticodon. The unique reference ID is in parentheses. Red letters indicate base pair differences between the unknown tRNA and the closest related tRNA reference with a tRNAScan determined anticodon (AlaCGC). The thick, red circle indicates the insertion in the anticodon mentioned in the main text. Nuclear gene 1009 had multiple more closely related sequences but without definitive anticodons see Supp. Table 1 nuclear reference ID#s 964, 1005-1014. Structures presented represent the maximum free energy folding model using the program the program RNAfold (Lorenz, et al. 2011) and diagram was created using the program VARNA ver. 3.9 (Darty, et al. 2009).

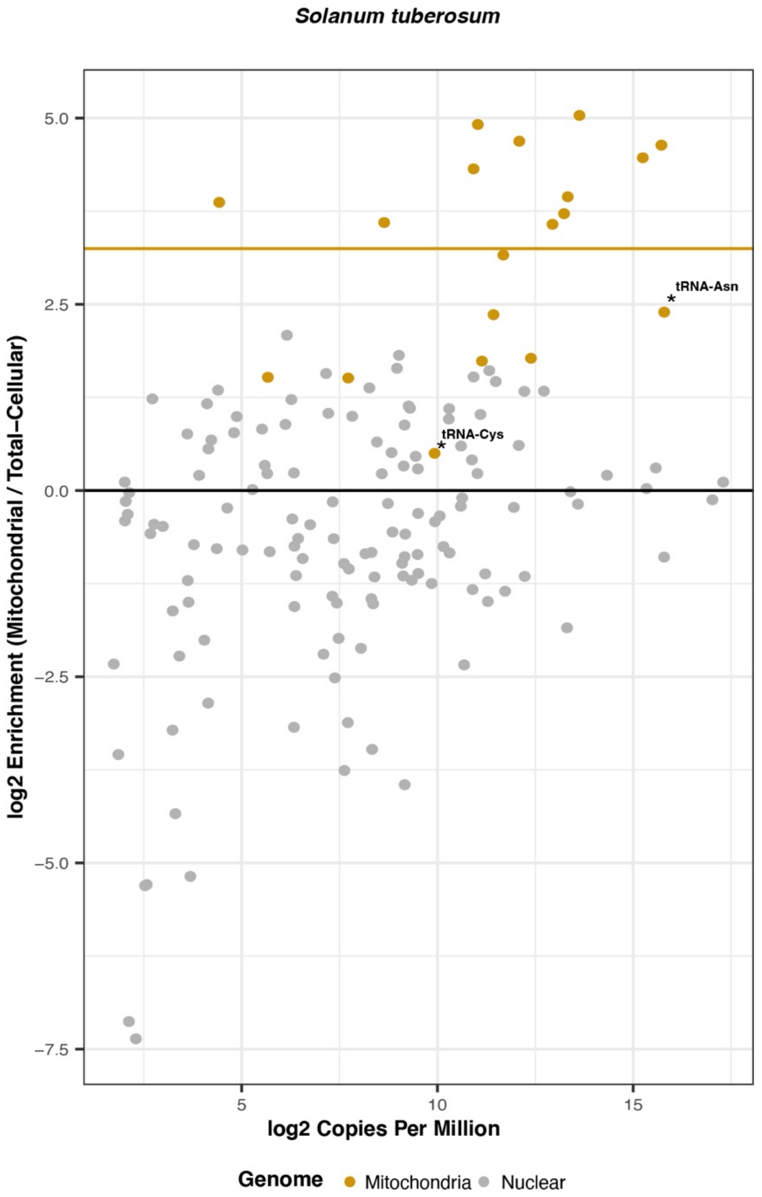

Supp. Fig. 5| Enrichment of mitochondrially encoded and nuclear-encoded tRNAs in the mitochondrial isolate relative to total-cellular sample in *S. tuberosum*. Each dot represents a unique tRNA sequence. The heavy gold line represents the average enrichment of mitochondrial-encoded genes. The genomic origin of the tRNA is indicated by color, with gray being nuclear-encoded and gold being mitochondrial. The two labeled mt-tRNAs (Cys and Asn) are identical in sequence to the plastid-encoded tRNA counterparts.

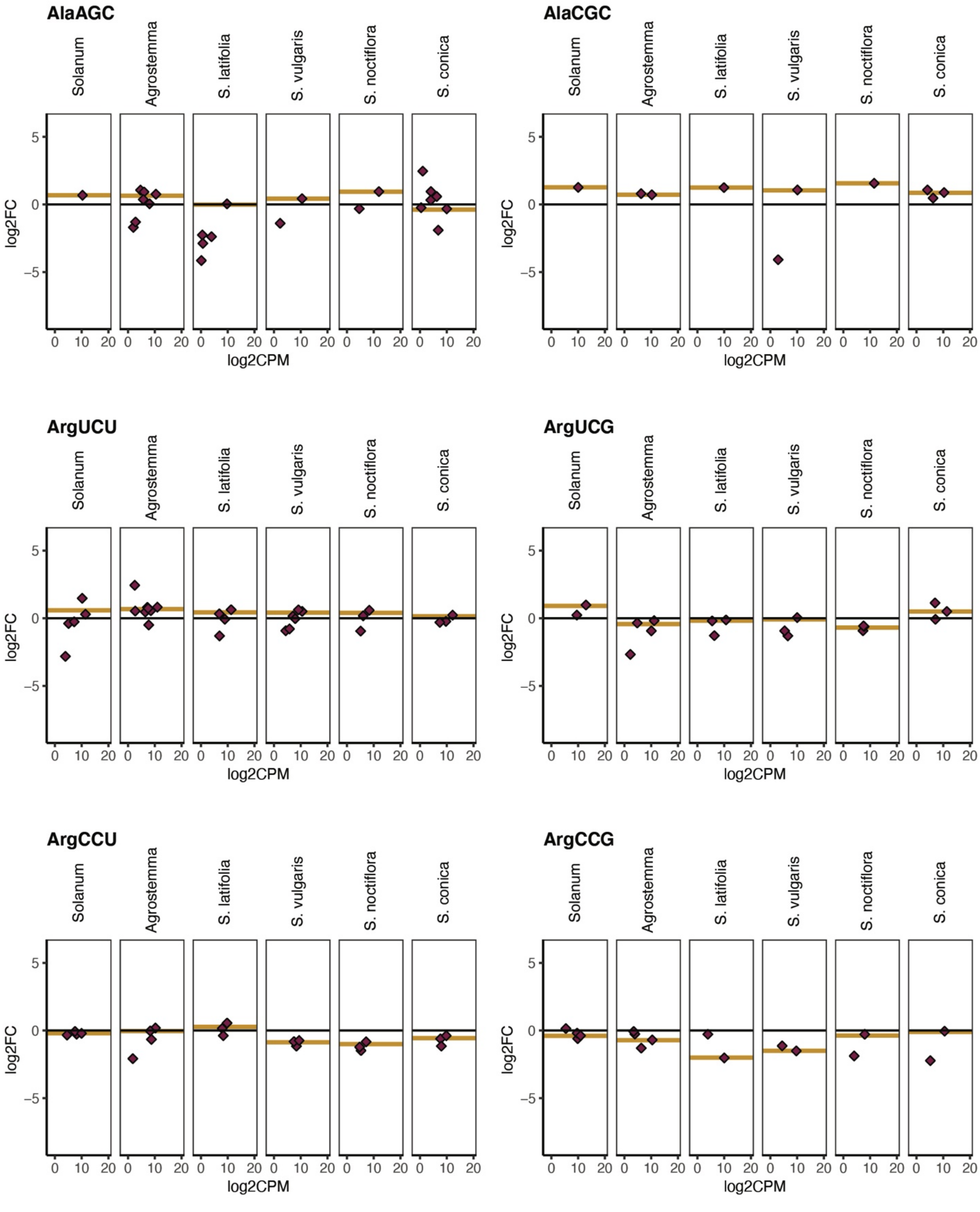
AlaAGC
AlaCGC
ArgUCU
ArgUCG
ArgCCU
ArgCCG
Solanum
Agrostemma
S. latifolia
S. vulgaris
S. noctiflora
S. conica
log2FC
log2CPM
5
0
−5
0
10
20

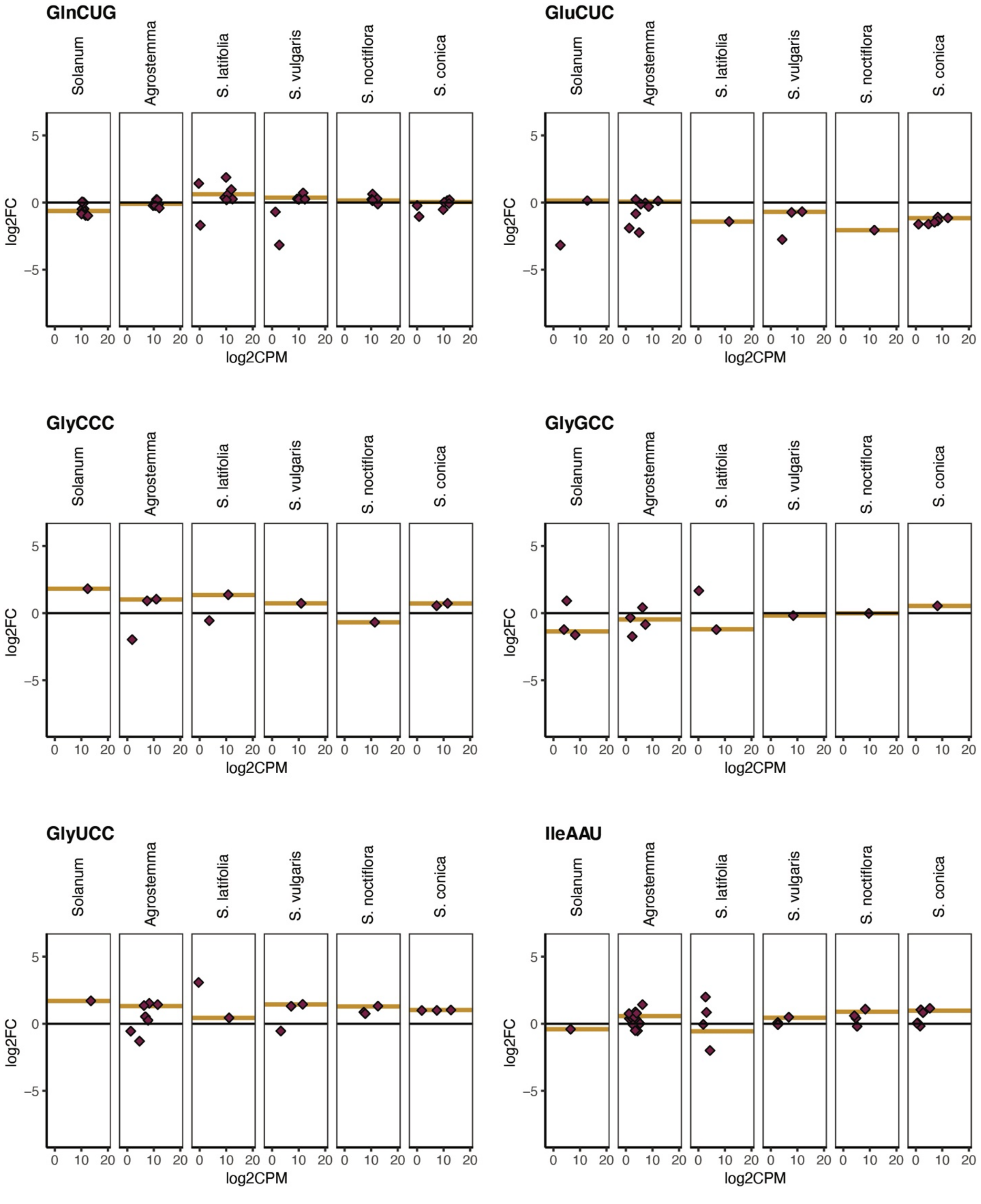
GlnCUG
GluCUC
GlyCCC
GlyGCC
GlyUCC
IleAAU
Solanum
Agrostemma
S. latifolia
S. vulgaris
S. noctiflora
S. conica
log2FC
log2CPM
5
0
−5
0
10
20

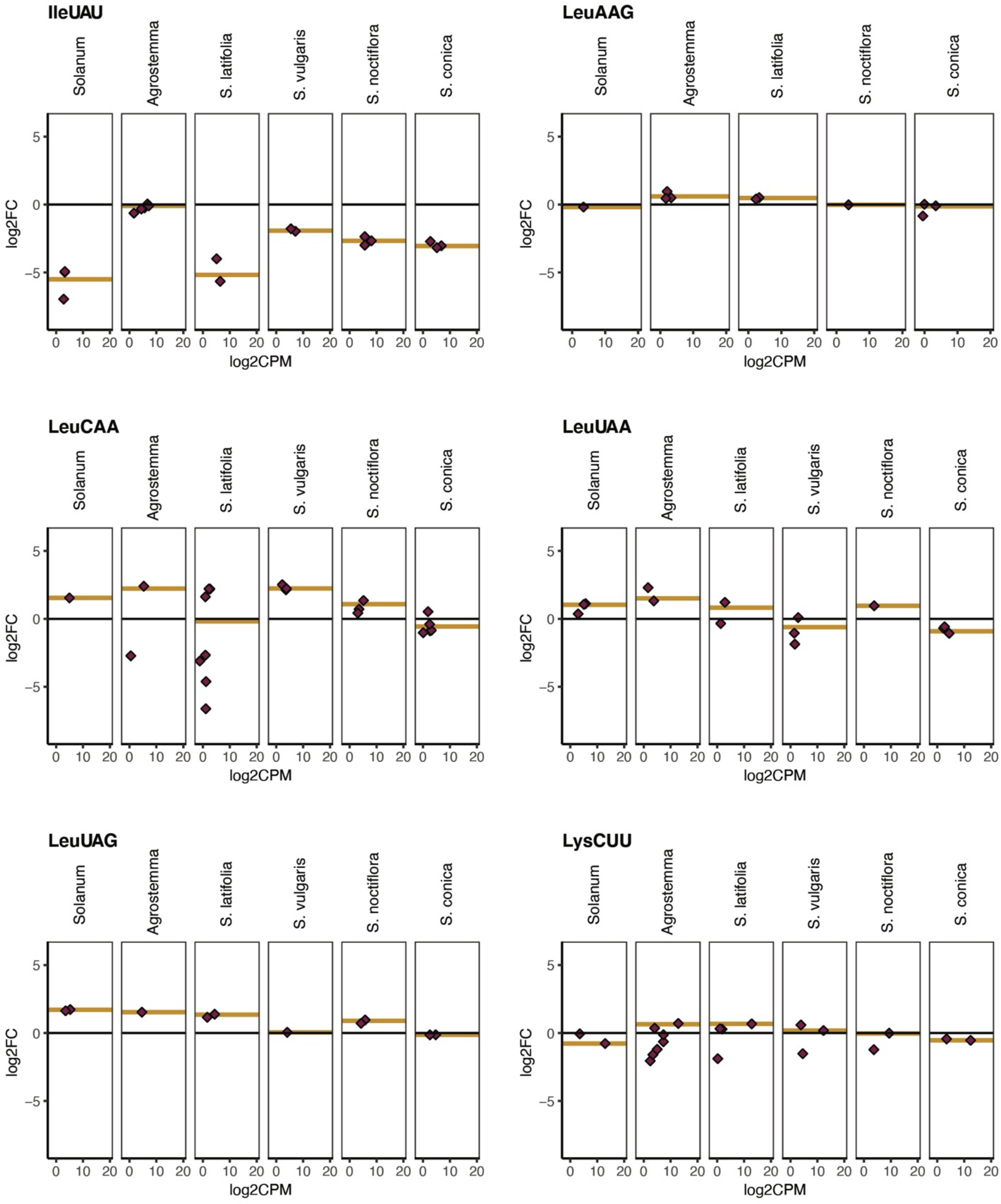
IleUAU
LeuAAG
LeuCAA
LeuUAA
LeuUAG
LysCUU
Solanum
Agrostemma
S. latifolia
S. vulgaris
S. noctiflora
S. conica
log2FC
log2CPM
5
0
−5
0
10
20

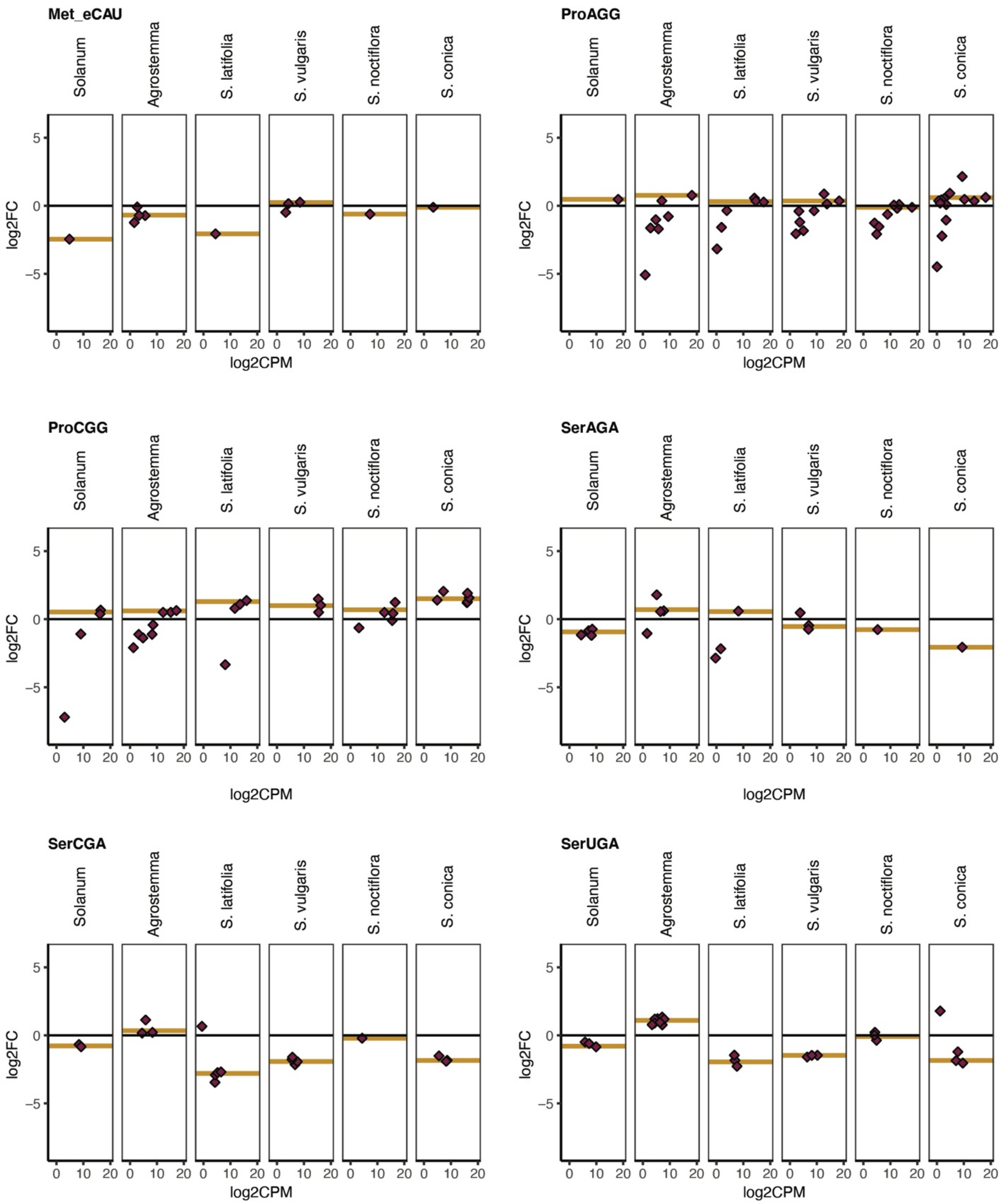
Met_eCAU
ProAGG
ProCGG
SerAGA
SerCGA
SerUGA
Solanum
Agrostemma
S. latifolia
S. vulgaris
S. noctiflora
S. conica
log2FC
log2CPM
5
0
−5
0
10
20

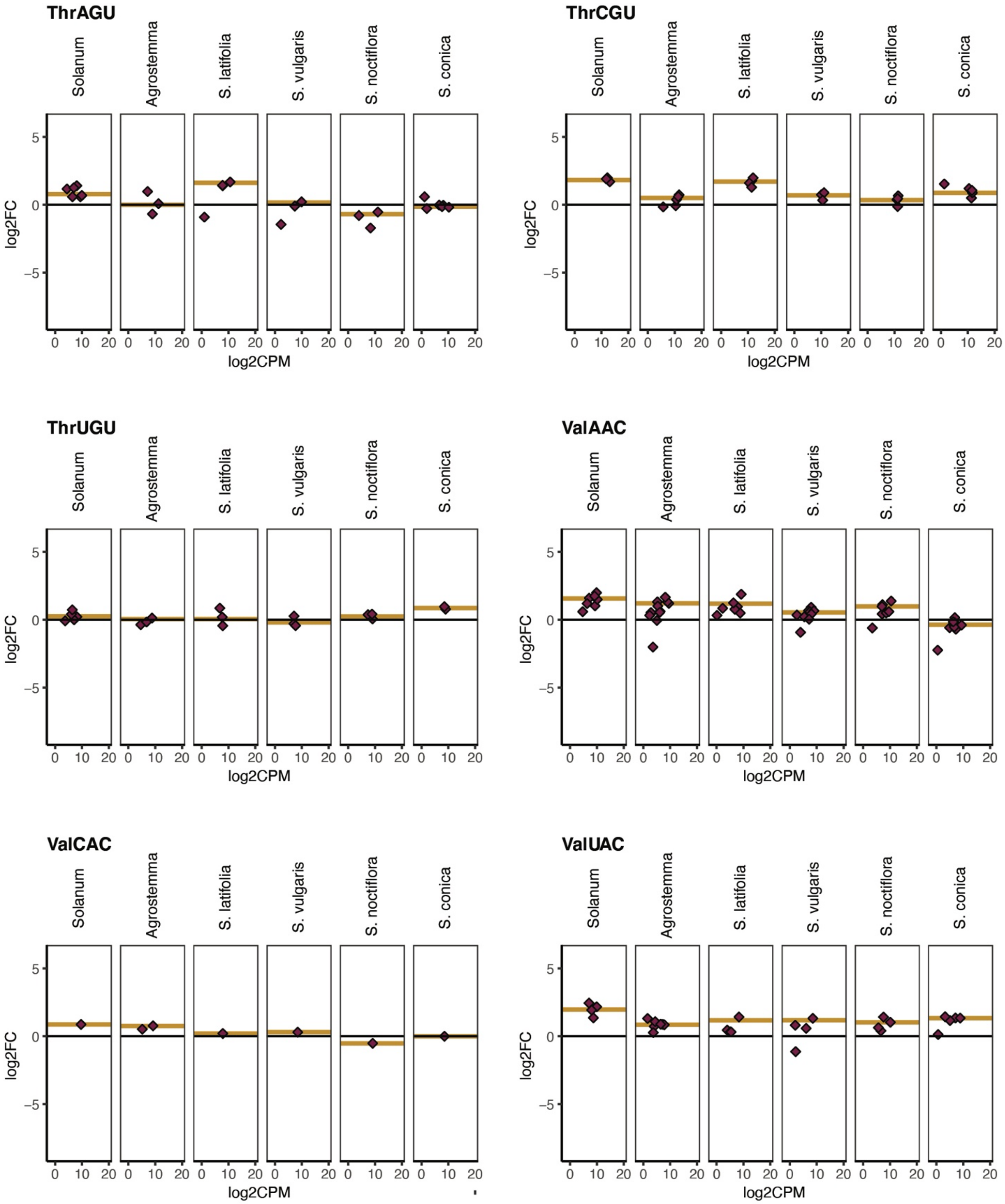

Supp. Fig. 6 | Enrichment and expression of individual cytosolic tRNA genes. The y-axis is enrichment in $\log_2$ fold change in mitochondrial isolates versus total-cellular samples. The x-axis is expression level in counts per million on a $\log_2$ scale. Points represent individual reference sequences, and gold lines represent the average enrichment of all nuclear-encoded tRNAs with that anticodon weighted by expression (see Methods).
